# Supplementary material for: Structures of honeybee-infecting Lake Sinai virus reveal domain functions and capsid assembly with dynamic motions
Source: Nat Commun. 2023 Feb 1;14:545. doi: 10.1038/s41467-023-36235-3 (PMC9892032; doi:10.1038/s41467-023-36235-3)
Supplement: Supplementary file 1 — Supplementary Information [file 41467_2023_36235_MOESM1_ESM.pdf]

Supplementary information

for

Structures of honeybee-infecting Lake Sinai virus reveal domain functions and capsid assembly with dynamic motions

Nai-Chi Chen[1#], Chun-Hsiung Wang[2#], Masato Yoshimura[1], Yi-Qi Yeh[1], Hong-Hsiang Guan[1], Phimonphan Chuankhayan[1], Chien-Chih Lin[1], Pei-Ju Lin[1,3], Yen-Chieh Huang[1], Soichi Wakatsuki[4], Meng-Chiao Ho[2*], Chun-Jung Chen[1,5,6,7*]

[1]Life Science Group, Scientific Research Division, National Synchrotron Radiation Research Center, Hsinchu, 30076, Taiwan

[2]Institute of Biological Chemistry, Academia Sinica, Taipei 115, Taiwan

[3]Institute of Bioinformatics and Structural Biology, National Tsing Hua University, Hsinchu 30043, Taiwan

[4]Department of Structural Biology, Stanford University, Stanford, CA 94305, U.S.A.

[5]Department of Physics, National Tsing Hua University, Hsinchu 30043, Taiwan

[6]Department of Biotechnology and Bioindustry Sciences, National Cheng Kung University, Tainan 701, Taiwan

[7]Department of Biological Science and Technology, National Yang Ming Chiao Tung University, Hsinchu 30010, Taiwan

Table of Contents

Figure 1–19
Table 1–8

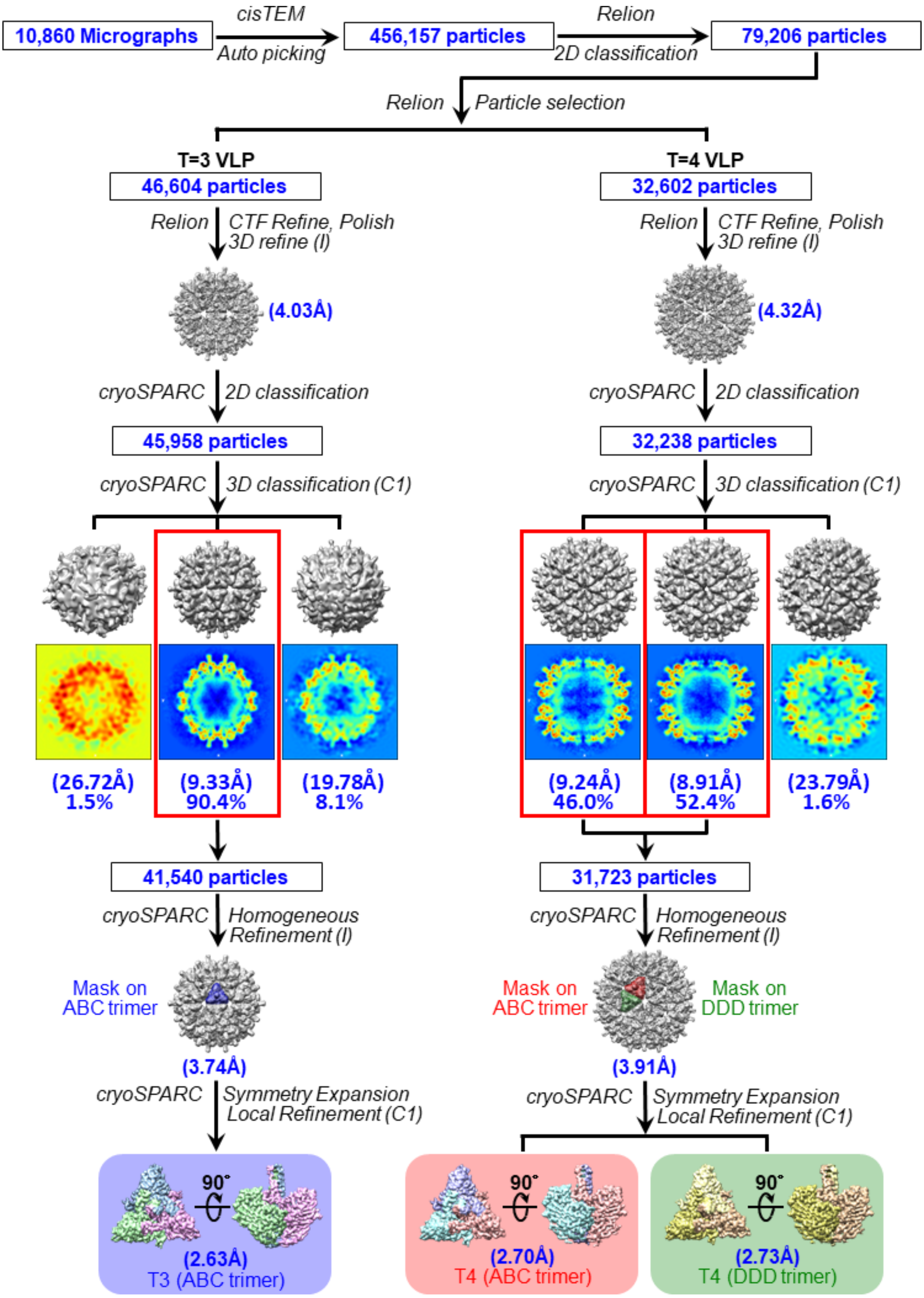

Fig. 1. The flow chart for cryo-EM data processing and structure determination of the LSV2 VLP at pH 6.5. Details are described in the Methods.

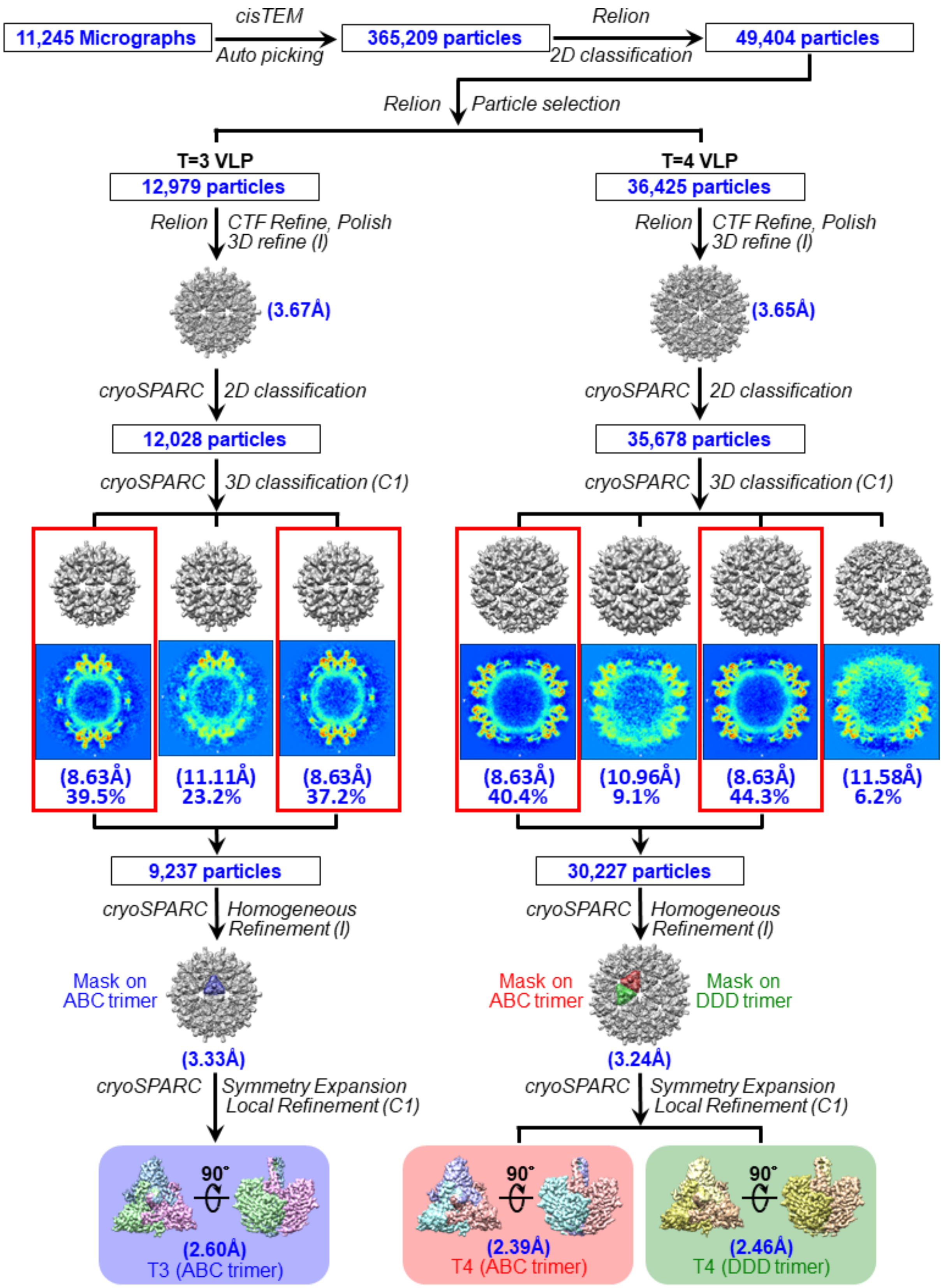

Fig. 2. The flow chart for cryo-EM data processing and structure determination of the LSV2 VLP at pH 7.5. Details are described in the Methods.

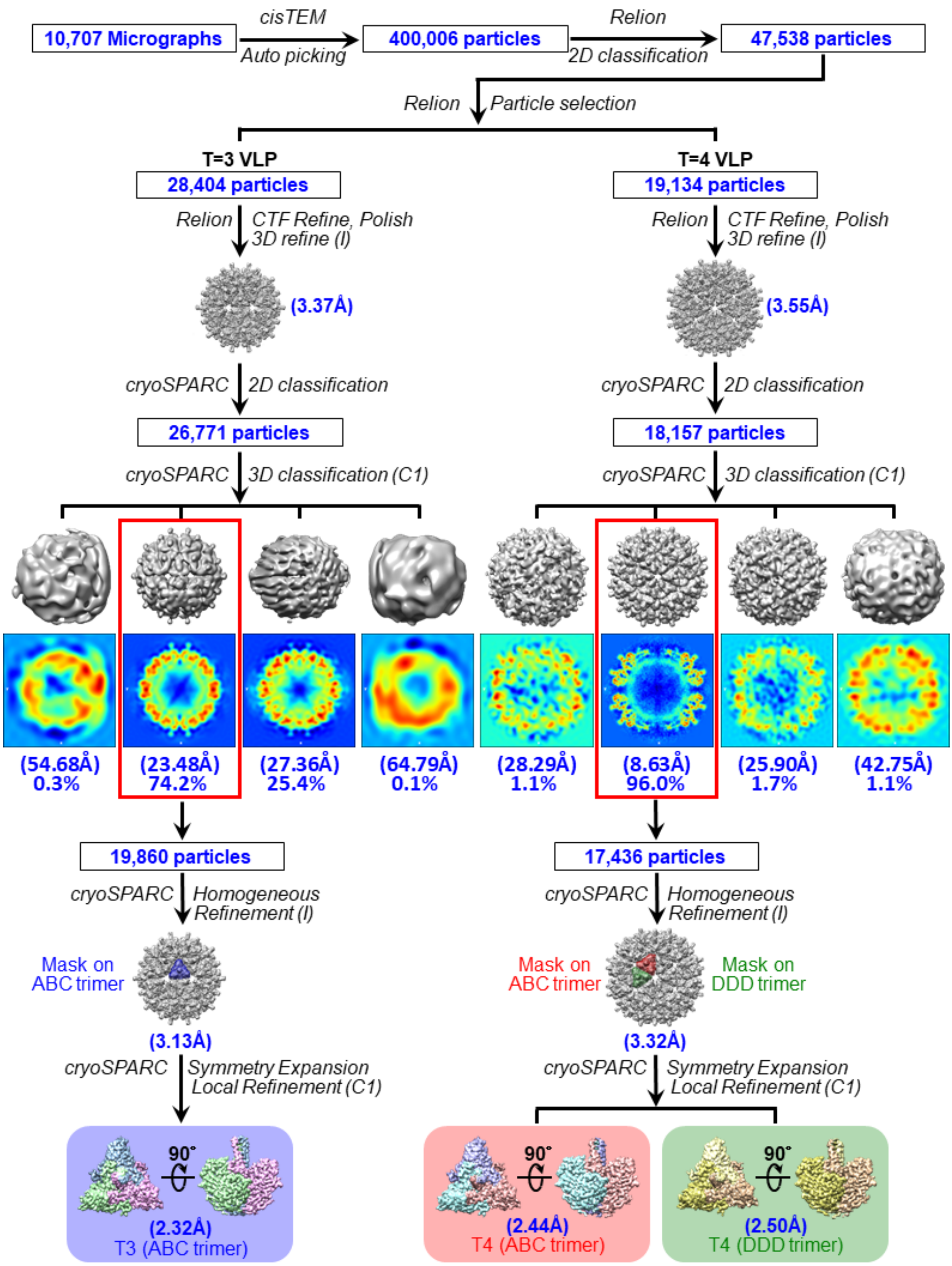

Fig. 3. The flow chart for cryo-EM data processing and structure determination of the LSV2 VLP at pH 8.5. Details are described in the Methods.

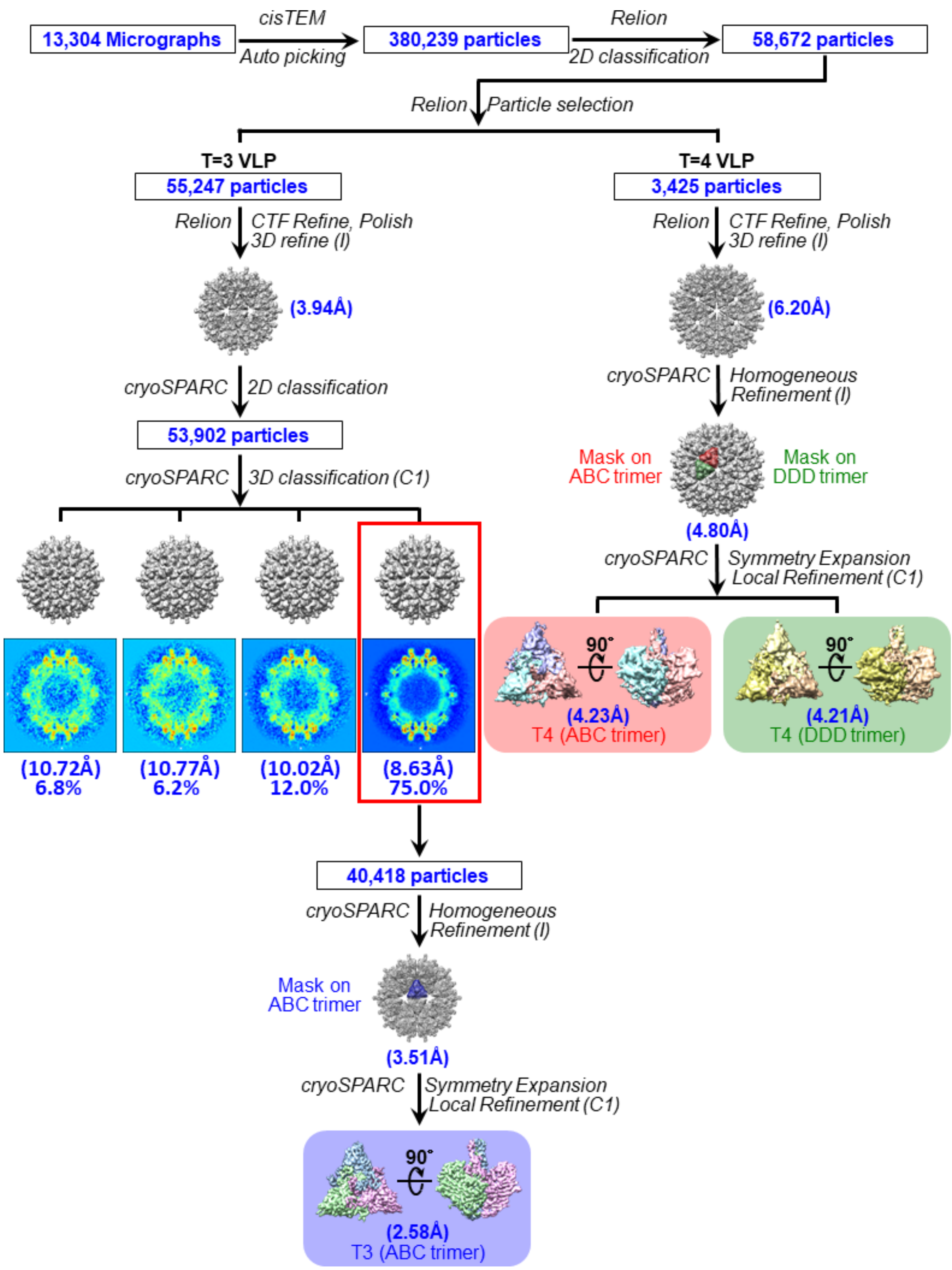

Fig. 4. The flow chart for cryo-EM data processing and structure determination of the delta-N48 LSV1 VLP at pH 6.5. Details are described in the Methods.

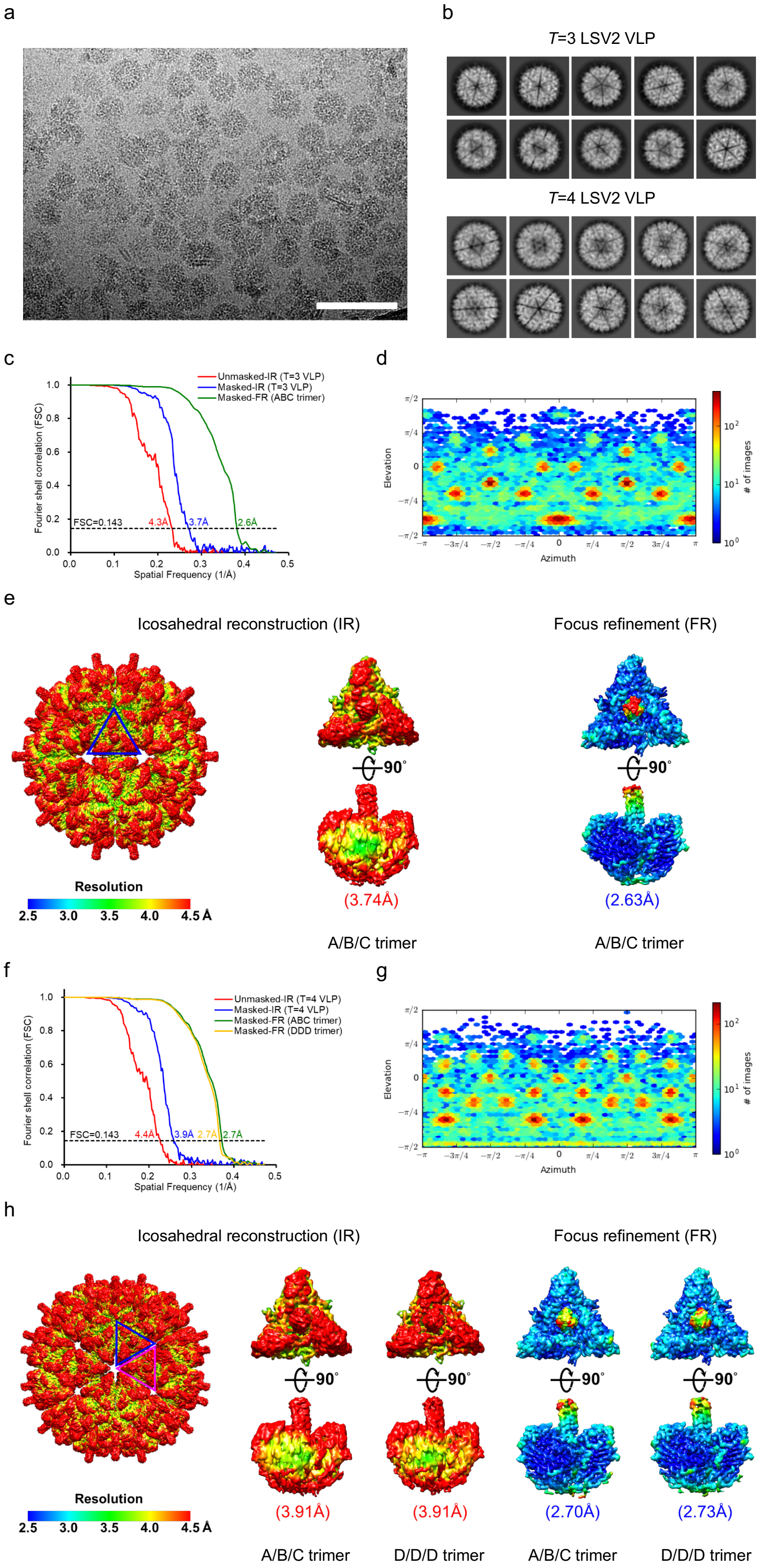
a
b
T=3 LSV2 VLP
T=4 LSV2 VLP
c
Unmasked-IR (T=3 VLP)
Masked-IR (T=3 VLP)
Masked-FR (ABC trimer)
Fourier shell correlation (FSC)
FSC=0.143
4.3Å
3.7Å
2.6Å
Spatial Frequency (1/Å)
d
Elevation
Azimuth
# of images
e
Icosahedral reconstruction (IR)
Focus refinement (FR)
90°
Resolution
2.5 3.0 3.5 4.0 4.5 Å
(3.74Å)
(2.63Å)
A/B/C trimer
A/B/C trimer
f
Unmasked-IR (T=4 VLP)
Masked-IR (T=4 VLP)
Masked-FR (ABC trimer)
Masked-FR (DDD trimer)
Fourier shell correlation (FSC)
FSC=0.143
4.4Å
3.9Å
2.7Å
2.7Å
Spatial Frequency (1/Å)
g
Elevation
Azimuth
# of images
h
Icosahedral reconstruction (IR)
Focus refinement (FR)
90°
Resolution
2.5 3.0 3.5 4.0 4.5 Å
(3.91Å)
(3.91Å)
(2.70Å)
(2.73Å)
A/B/C trimer
D/D/D trimer
A/B/C trimer
D/D/D trimer

Fig. 5. Cryo-EM reconstruction of LSV2 VLP at pH 6.5. a The representative motion-corrected and dose-weighted cryo-electron micrograph of frozen hydrated LSV2 VLP at pH 6.5. Bar: 100 nm. b The representative 2D class averages highlight the good alignment of the *T*=3 (upper) and *T*=4 (lower) VLPs. c FSC curves of the 3D reconstruction of unmasked (red) and masked (blue) *T*=3 VLP with icosahedral symmetry imposed. The FSC curve of focused refinement on A/B/C (green) trimer is also shown. FSC 0.143 criterion was used for resolution determination. d The relative angular distribution of all particles in the final 3D reconstruction calculated in cryoSPARC. The heat map is colored by estimates of the number of particles per view. Red represents a view with more particles, whereas blue with less. e Surface view of the density map of *T*=3 VLP and the segmented A/B/C trimer (blue triangular) at a resolution of 3.74 Å. The resolution was further improved to 2.63 Å after focused refinement on A/B/C trimer. The cryo-EM map is colored according to the local resolution. f FSC curves of the 3D reconstruction of unmasked (red) and masked (blue) *T*=4 VLP with icosahedral symmetry imposed. The FSC curve of focused refinement on A/B/C (green) and D/D/D (yellow) trimer is also shown. g The relative angular distribution of all particles in the final 3D reconstruction. The heat map is colored by estimates of the number of particles per view. Red represents a view with more particles, whereas blue with less. h Surface view of the density map of the *T*=4 VLP and the segmented A/B/C and D/D/D trimer at a resolution of 3.91 Å. The resolution was further improved to 2.70 and 2.73 Å after focused refinement on A/B/C and D/D/D trimer, respectively. The cryo-EM map is colored according to the local resolution.

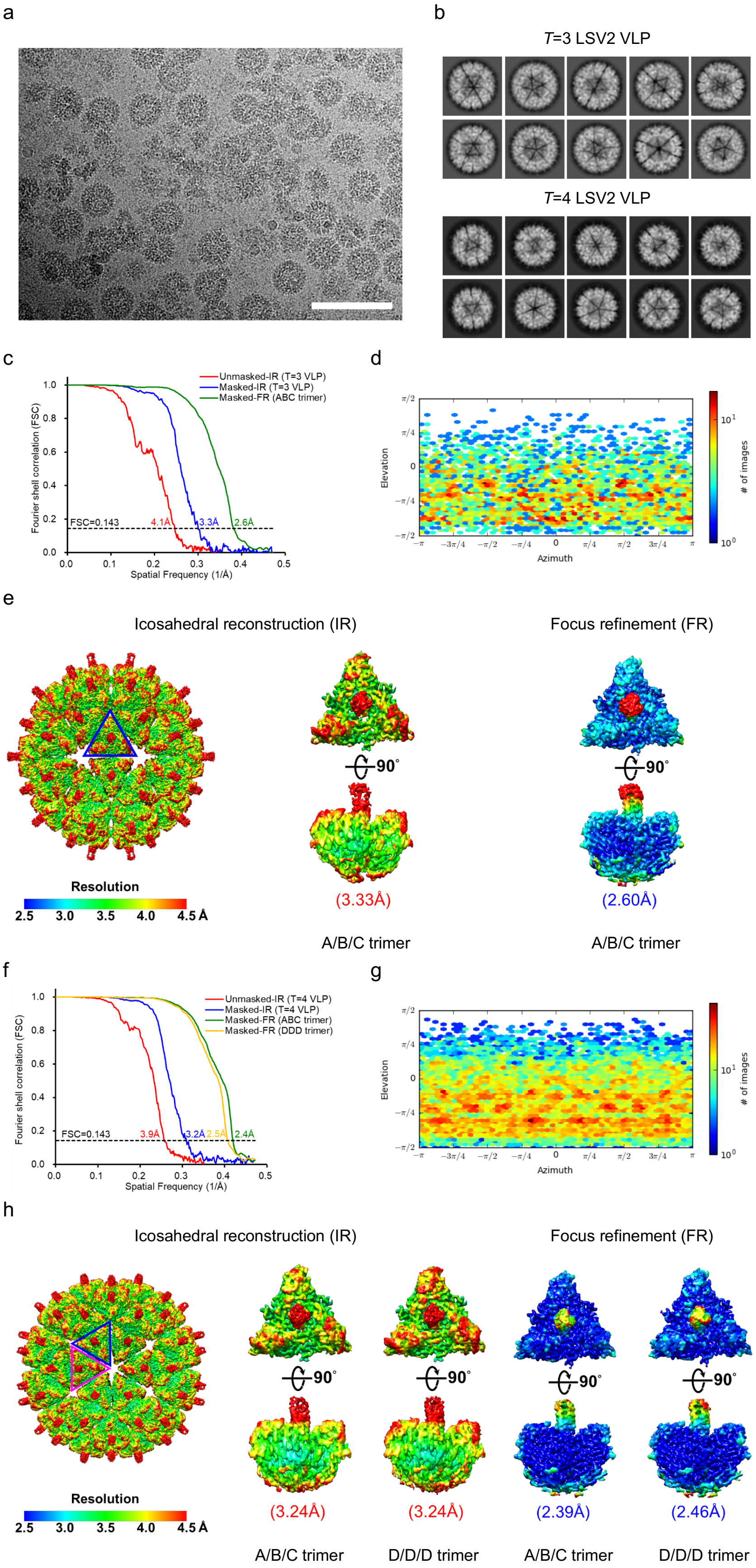
a
b
T=3 LSV2 VLP
T=4 LSV2 VLP
c
Unmasked-IR (T=3 VLP)
Masked-IR (T=3 VLP)
Masked-FR (ABC trimer)
Fourier shell correlation (FSC)
FSC=0.143
4.1Å
3.3Å
2.6Å
Spatial Frequency (1/Å)
d
Elevation
Azimuth
# of images
e
Icosahedral reconstruction (IR)
Focus refinement (FR)
90°
Resolution
2.5 3.0 3.5 4.0 4.5 Å
(3.33Å)
(2.60Å)
A/B/C trimer
A/B/C trimer
f
Unmasked-IR (T=4 VLP)
Masked-IR (T=4 VLP)
Masked-FR (ABC trimer)
Masked-FR (DDD trimer)
Fourier shell correlation (FSC)
FSC=0.143
3.9Å
3.2Å
2.5Å
2.4Å
Spatial Frequency (1/Å)
g
Elevation
Azimuth
# of images
h
Icosahedral reconstruction (IR)
Focus refinement (FR)
90°
Resolution
2.5 3.0 3.5 4.0 4.5 Å
(3.24Å)
(3.24Å)
(2.39Å)
(2.46Å)
A/B/C trimer
D/D/D trimer
A/B/C trimer
D/D/D trimer

Fig. 6. Cryo-EM reconstruction of LSV2 VLP at pH 7.5. a The representative motion-corrected and dose-weighted cryo-electron micrograph of frozen hydrated LSV2 VLP at pH 7.5. Bar: 100 nm. b The representative 2D class averages highlight the good alignment of the *T*=3 (upper) and *T*=4 (lower) VLPs. c FSC curves of the 3D reconstruction of unmasked (red) and masked (blue) *T*=3 VLP with icosahedral symmetry imposed. The FSC curve of focused refinement on A/B/C (green) trimer is also shown. FSC 0.143 criterion was used for resolution determination. d The relative angular distribution of all particles in the final 3D reconstruction calculated in cryoSPARC. The heat map is colored by estimates of the number of particles per view. Red represents a view with more particles, where blue with less. e Surface view of the density map of the *T*=3 VLP and the segmented A/B/C trimer (blue triangular) at a resolution of 3.33 Å. The resolution was further improved to 2.60 Å after focused refinement on A/B/C trimer. The cryo-EM map is colored according to the local resolution. f FSC curves of the 3D reconstruction of unmasked (red) and masked (blue) *T*=4 VLP with icosahedral symmetry imposed. The FSC curve of focused refinement on A/B/C (green) and D/D/D (yellow) trimer is also shown. g The relative angular distribution of all particles in the final 3D reconstruction. The heat map is colored by estimates of the number of particles per view. Red represents a view with more particles, whereas blue with less. h Surface view of the density map of the *T*=4 VLP and the segmented A/B/C (blue triangular) and D/D/D (pink triangular) trimer at a resolution of 3.24 Å. The resolution was improved to 2.39 Å and 2.46 Å after focused refinement on A/B/C and D/D/D trimer, respectively. The cryo-EM map is colored according to the local resolution.

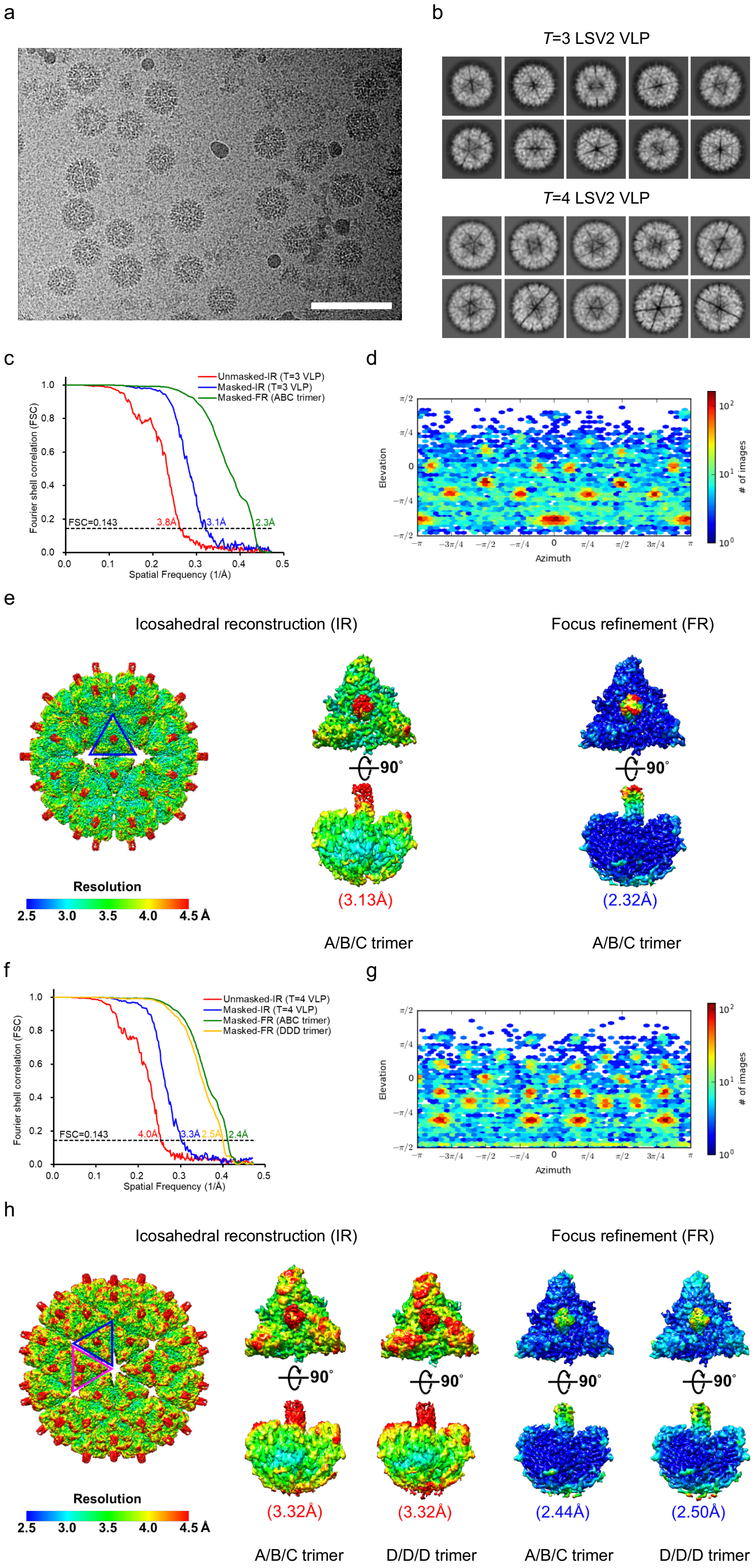

a
b
T=3 LSV2 VLP
T=4 LSV2 VLP
c
Unmasked-IR (T=3 VLP)
Masked-IR (T=3 VLP)
Masked-FR (ABC trimer)
Fourier shell correlation (FSC)
FSC=0.143
3.8Å
3.1Å
2.3Å
Spatial Frequency (1/Å)
d
Elevation
Azimuth
# of images
e
Icosahedral reconstruction (IR)
Focus refinement (FR)
90°
Resolution
2.5 3.0 3.5 4.0 4.5 Å
(3.13Å)
(2.32Å)
A/B/C trimer
A/B/C trimer
f
Unmasked-IR (T=4 VLP)
Masked-IR (T=4 VLP)
Masked-FR (ABC trimer)
Masked-FR (DDD trimer)
Fourier shell correlation (FSC)
FSC=0.143
4.0Å
3.3Å
2.5Å
2.4Å
Spatial Frequency (1/Å)
g
Elevation
Azimuth
# of images
h
Icosahedral reconstruction (IR)
Focus refinement (FR)
90°
Resolution
2.5 3.0 3.5 4.0 4.5 Å
(3.32Å)
(3.32Å)
(2.44Å)
(2.50Å)
A/B/C trimer
D/D/D trimer
A/B/C trimer
D/D/D trimer

Fig. 7. Cryo-EM reconstruction of LSV2 VLP at pH 8.5. a The representative motion-corrected and dose-weighted cryo-electron micrograph of frozen hydrated LSV2 VLP at pH 8.5. Bar: 100 nm. b The representative 2D class averages highlight the good alignment of the *T*=3 (upper) and *T*=4 (lower) VLPs. c FSC curves of the 3D reconstruction of unmasked (red) and masked (blue) *T*=3 VLP with icosahedral symmetry imposed. The FSC curve of focused refinement on A/B/C (green) trimer is also shown. FSC 0.143 criterion was used for resolution determination. d The relative angular distribution of all particles in the final 3D reconstruction calculated in cryoSPARC. The heat map is colored by estimates of the number of particles per view. Red represents a view with more particles, where blue with less. e Surface view of the density map of the *T*=3 VLP and the segmented A/B/C trimer (blue triangular) at a resolution of 3.13 Å. The resolution was improved to 2.32 Å after focused refinement on A/B/C trimer. The cryo-EM map is colored according to the local resolution. f FSC curves of the 3D reconstruction of unmasked (red) and masked (blue) *T*=4 VLP with icosahedral symmetry imposed. The FSC curves of focused refinement on A/B/C (green) and D/D/D (yellow) trimer are also shown. g The relative angular distribution of all particles in the final 3D reconstruction. The heat map is colored by estimates of the number of particles per view. Red represents a view with more particles, whereas blue with less. h Surface view of the density map of the *T*=4 VLP and the segmented A/B/C (blue triangular) and D/D/D (pink triangular) trimer at a resolution of 3.32 Å. The resolution was improved to 2.44 Å and 2.50 Å after focused refinement on A/B/C and D/D/D trimer, respectively. The cryo-EM map is colored according to the local resolution.

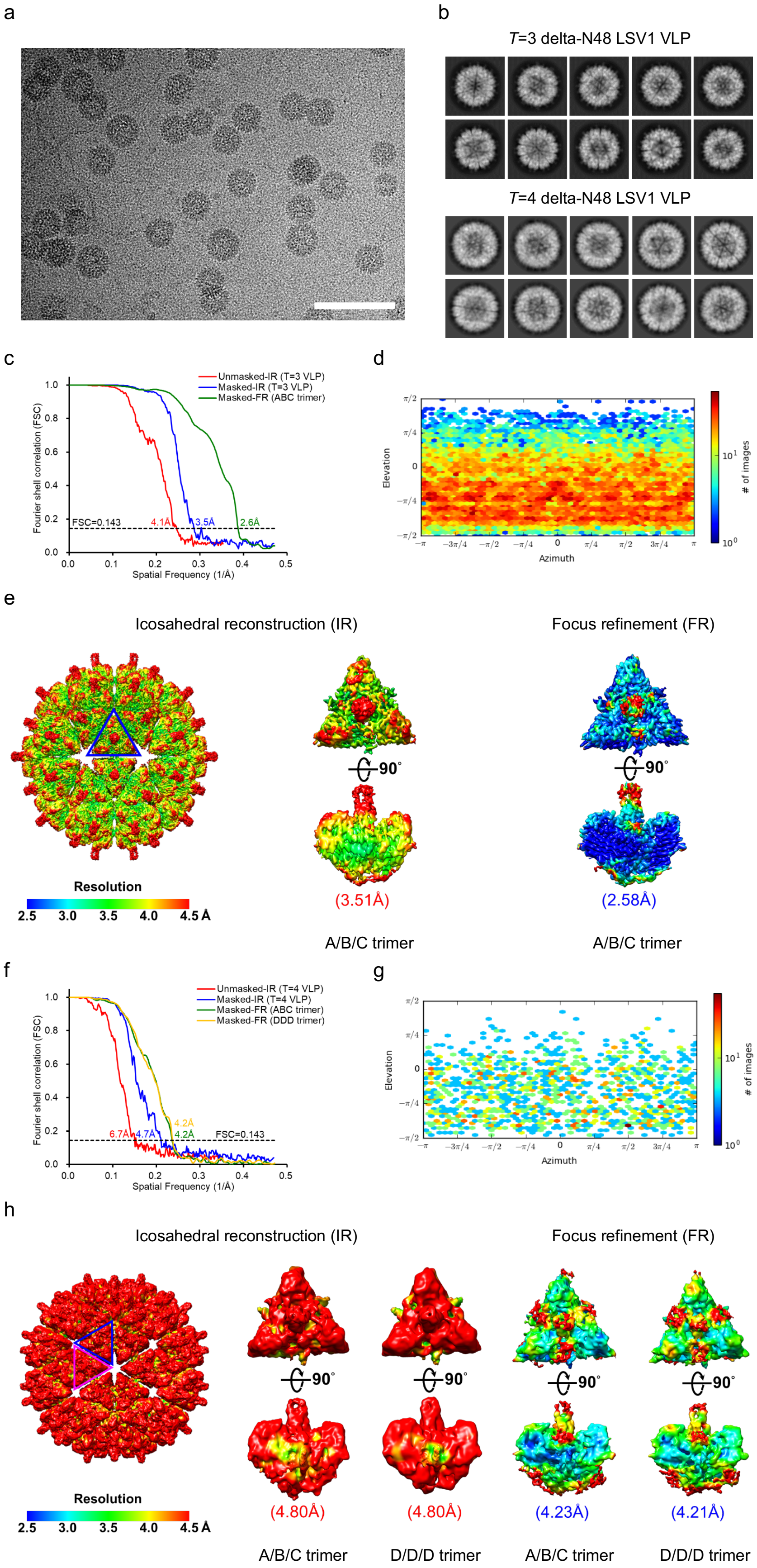

a
b
T=3 delta-N48 LSV1 VLP
T=4 delta-N48 LSV1 VLP
c
Unmasked-IR (T=3 VLP)
Masked-IR (T=3 VLP)
Masked-FR (ABC trimer)
Fourier shell correlation (FSC)
FSC=0.143
4.1Å
3.5Å
2.6Å
Spatial Frequency (1/Å)
d
Elevation
Azimuth
# of images
e
Icosahedral reconstruction (IR)
Focus refinement (FR)
90°
Resolution
2.5 3.0 3.5 4.0 4.5 Å
(3.51Å)
(2.58Å)
A/B/C trimer
A/B/C trimer
f
Unmasked-IR (T=4 VLP)
Masked-IR (T=4 VLP)
Masked-FR (ABC trimer)
Masked-FR (DDD trimer)
6.7Å
4.7Å
4.2Å
4.2Å
FSC=0.143
g
h
Icosahedral reconstruction (IR)
Focus refinement (FR)
(4.80Å)
(4.80Å)
(4.23Å)
(4.21Å)
A/B/C trimer
D/D/D trimer
A/B/C trimer
D/D/D trimer

Fig. 8. Cryo-EM reconstruction of delta-N48 LSV1 VLP at pH 6.5. a The representative motion-corrected and dose-weighted cryo-electron micrograph of frozen hydrated delta-N48 LSV1 VLP at pH 6.5. Bar: 100 nm. b The representative 2D class averages highlight the good alignment of the *T*=3 (upper) and *T*=4 (lower) VLPs. c FSC curves of the 3D reconstruction of unmasked (red) and masked (blue) *T*=3 VLP with icosahedral symmetry imposed. The FSC curve of focused refinement on A/B/C (green) trimer is also shown. FSC 0.143 criterion was used for resolution determination. d The relative angular distribution of all particles in the final 3D reconstruction calculated in cryoSPARC. The heat map is colored by estimates of the number of particles per view. Red represents a view with more particles, whereas blue with less. e Surface view of the density map of the *T*=3 VLP and the segmented A/B/C trimer (blue triangular) at a resolution of 3.51 Å. The resolution was improved to 2.58 Å after focused refinement on A/B/C trimer. The cryo-EM map is colored according to the local resolution. f FSC curves of the 3D reconstruction of unmasked (red) and masked (blue) *T*=4 VLP with icosahedral symmetry imposed. The FSC curves of focused refinement on A/B/C (green) and D/D/D (yellow) trimer are also shown. g The relative angular distribution of all particles in the final 3D reconstruction. The heat map is colored by estimates of the number of particles per view. Red represents a view with more particles, whereas blue with less. h Surface view of the density map of the *T*=4 VLP and the segmented A/B/C (blue triangular) and D/D/D (pink triangular) trimer at a resolution of 4.80 Å. The resolution was improved to 4.23 Å and 4.21 Å after focused refinement on A/B/C and D/D/D trimer, respectively. The cryo-EM map is colored according to the local resolution.

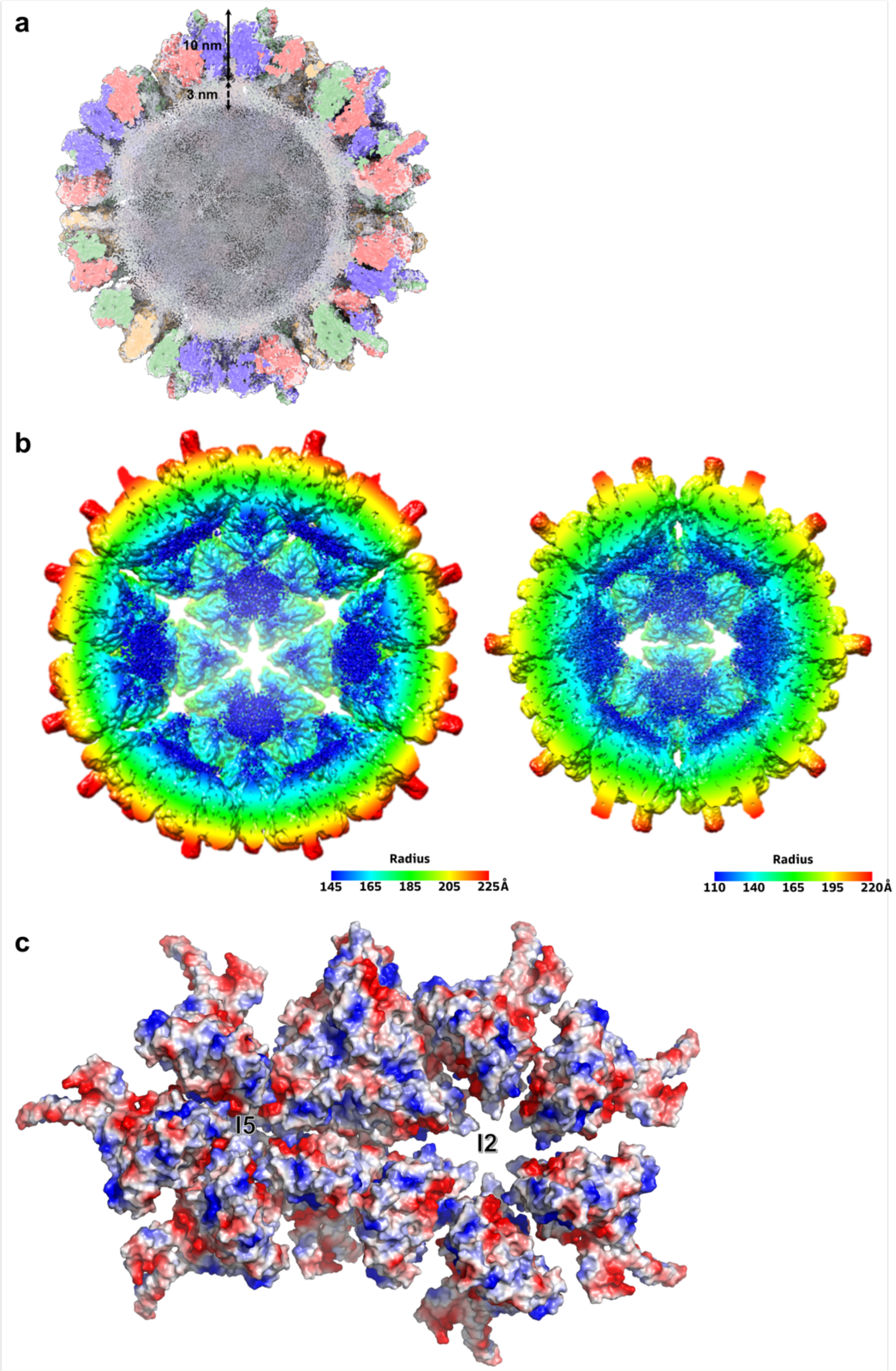

Fig. 9 Cryo-EM analysis of the *T*=4 and *T*=3 LSV2 and delta-N48 LSV1 VLPs. a A cutaway view of the cryo-EM map of *T*=4 LSV2 VLP with global refinement. The thickness of the protein shell is ~100 Å and the thickness of an additional electron-density layer is ~30 Å. b The cutaway views of the *T*=4 and *T*=3 LSV2 VLPs. Half-sectional views of the *T*=4 (left) and *T*=3 (right) LSV2 VLPs reveal extra average densities (blue) around inner surface at I5 axes. The 3D maps are colored by estimates of radius. c The molecular electrostatic potential inner surface of the *T*=4 LSV2 VLP. The inner surface with a bottom view along I5 and I2 axes is colored in red and blue for negatively and positively charged regions.

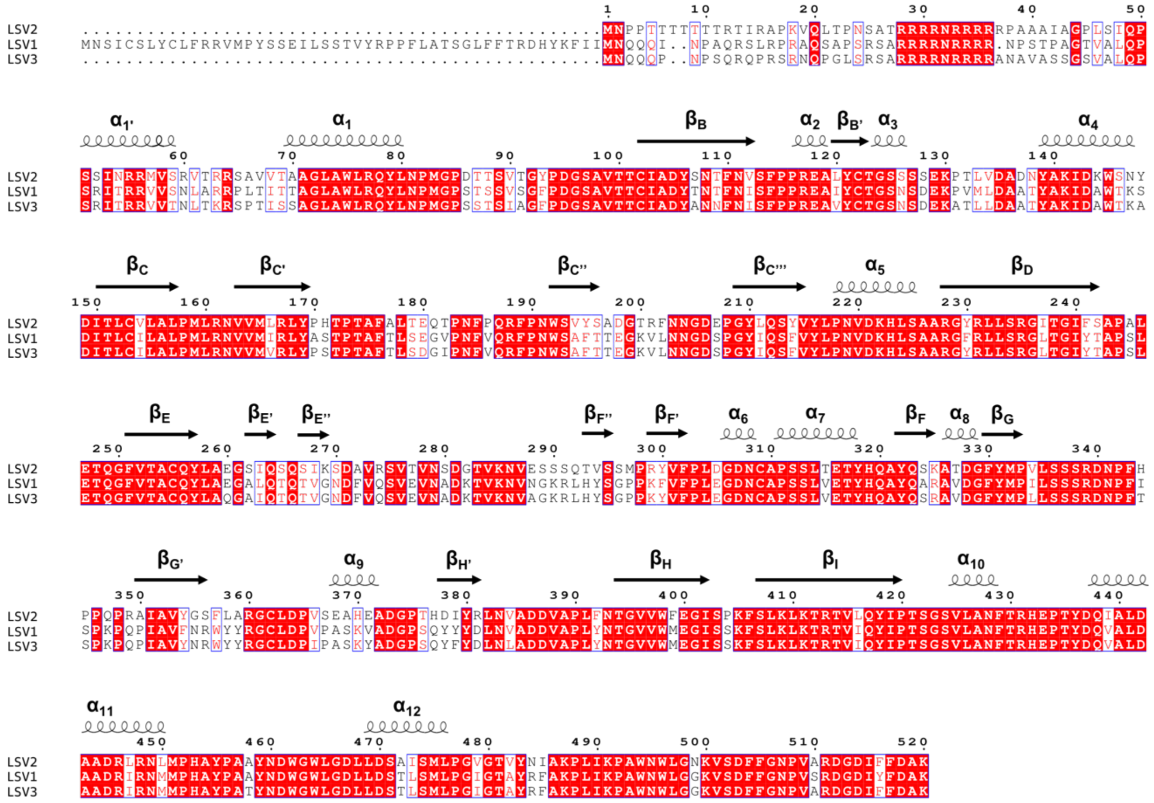

Fig. 10 Sequence alignment of RNA-encoded CPs of three LSV strains. Multiple sequence alignment was performed with sequences of the CPs from LSV1, LSV2 and LSV3 using ClustalW. Secondary structures and sequence numbers on the top refer to the CP structures of LSV2 and delta-N48 LSV1.

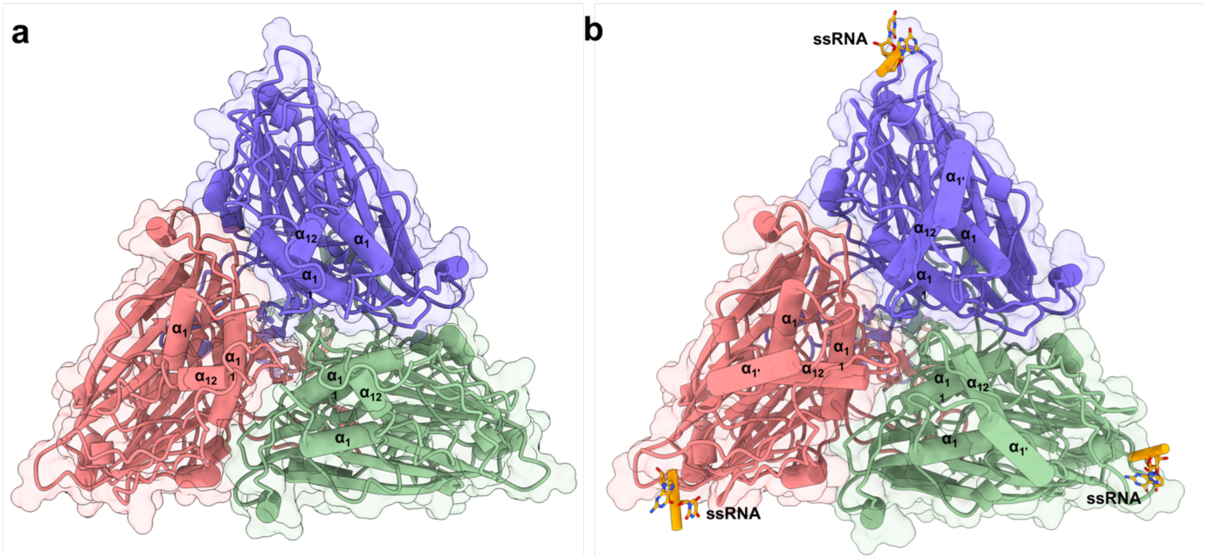

Fig. 11 Helical domains of three subunits A, B and C of *T*=4 LSV2 (a) and *T*=3 delta-N48 LSV1 (b) VLPs. Subunits A, B, C are shown in purple, red, green, respectively. ssRNAs are shown in orange.

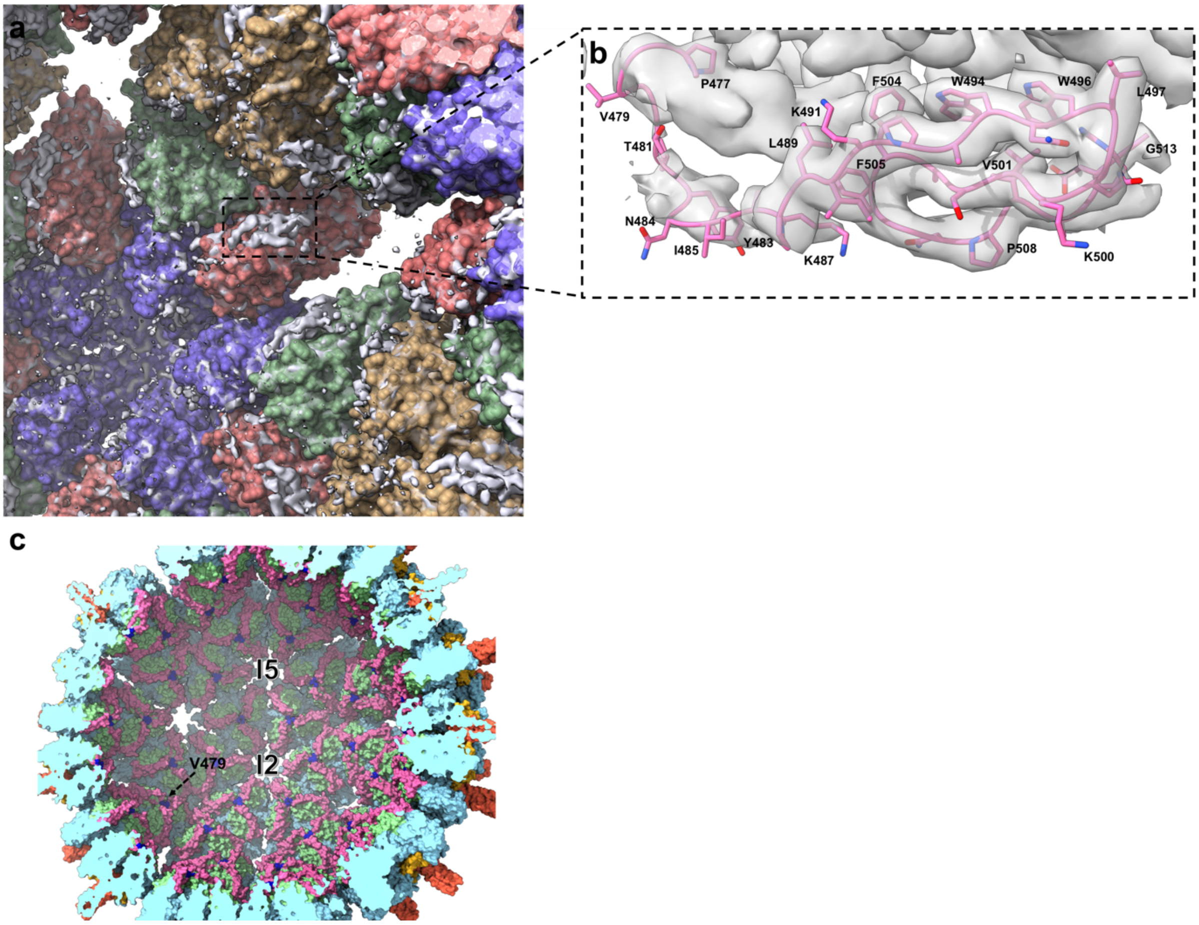

Fig. 12 Internal C-arms of *T*=4 LSV2 VLP. a C-arm structures (gray) of capsid subunits shown in a color rendering of the cryo-EM map of *T*=4 LSV2 VLP. 240 monomers arranged as pentameric and hexameric capsomeres colored in purple (subunit A), red (subunit B), green (subunit C), and yellow (subunit D), respectively, as in Fig. 1c. b Representative segments of the atomic model of C-arm (magenta) with the corresponding density (gray). c A cutaway view portion of the *T*=4 LSV2 VLP structure shows the C-arm (pink). The residues V479 from the C-arms are colored in blue. All subunits are colored as in Fig. 1d.

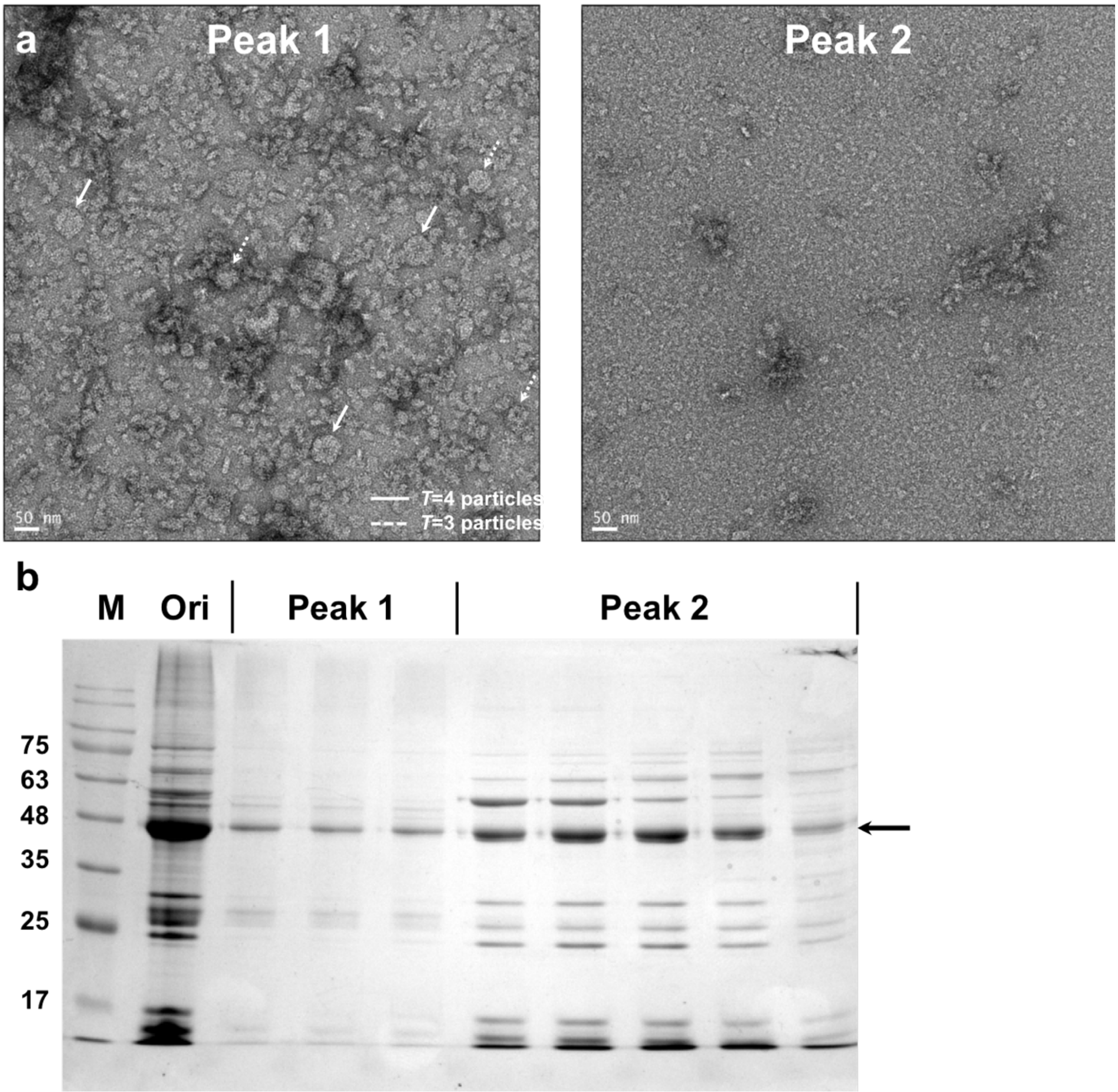

Fig. 13 Purified the anchor loop deletion of LSV2 CP assembled *in vitro* shows two major peaks by SEC. a Electron micrograph revealed the larger complexes and particles assembled *in vitro* from the Peak1 sample by SEC (*T*=4 and *T*=3 particles are indicated by white line and white dotted arrows, respectively) (left). Electron micrograph revealed the small CPs and intermediate from the Peak 2 sample by SEC (right). 20 independent micrographs gave similar results. b The SDS-PAGE gel showed a single band of CPs for the Peak 1 sample, whereas CPs as a major band assembled into intermediates and some minor contaminated proteins from *E. coli* for the Peak 2. Ori is the specimen of the anchor loop deletion of LSV2 CP after *in vitro* self-assembly.

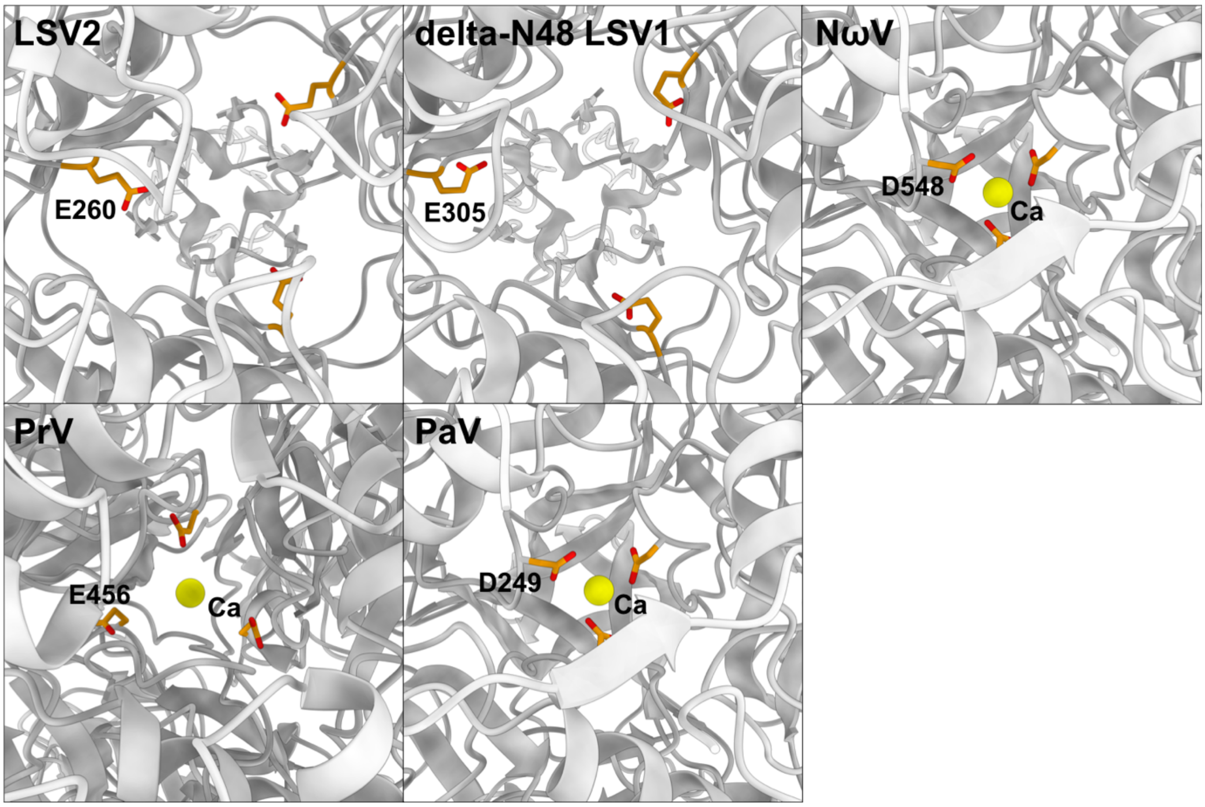

Fig. 14 Bottom views of the homo-trimeric capsomeres among the LSV2, delta-N48 LSV1, NωV, PrV and PaV. These three subunits A, B and C are colored in gray. All negatively charged residues and the calcium ion in these subunits are shown in orange sticks and yellow spheres, respectively.

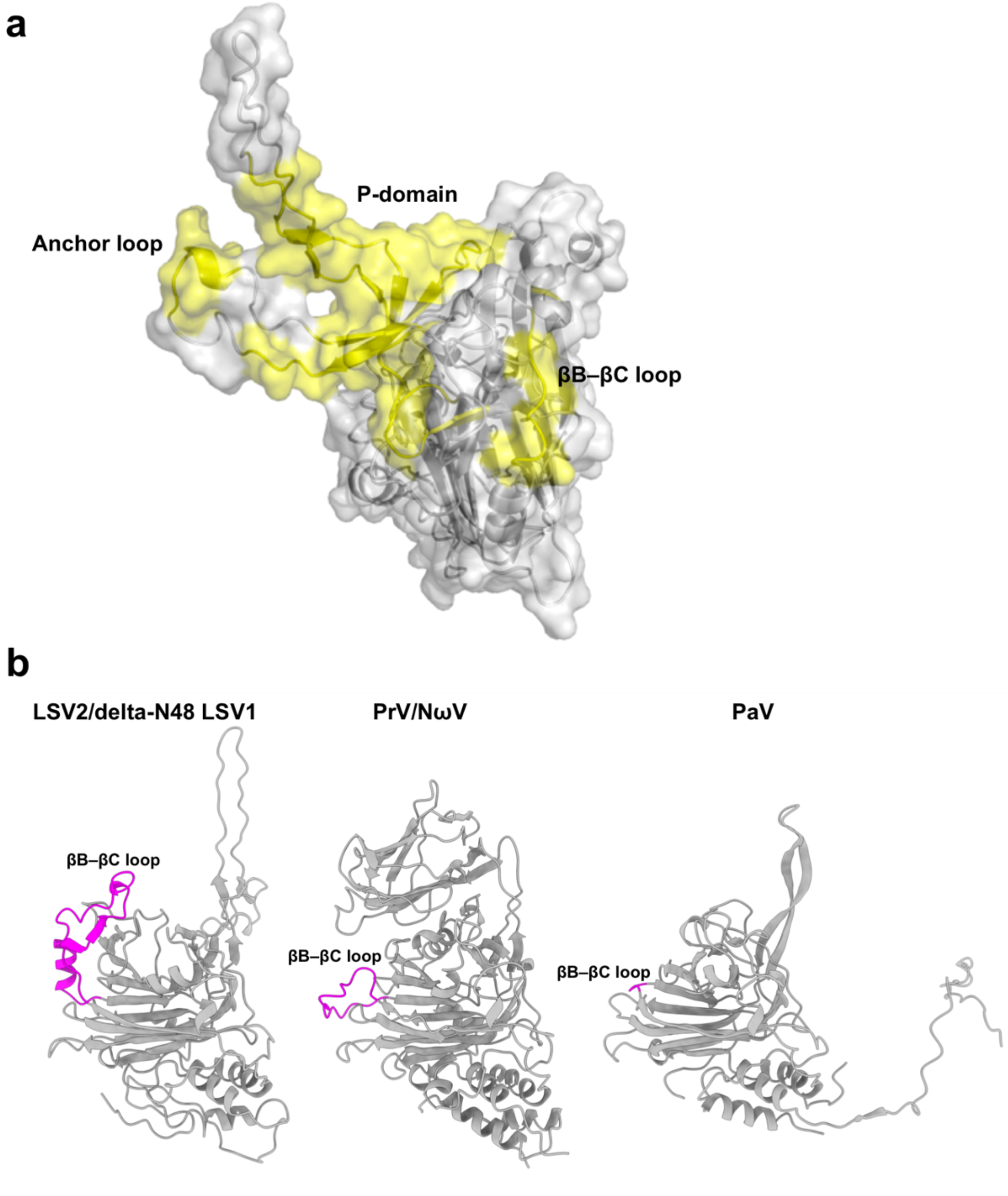

Fig. 15 The hypervariable regions of CPs among different strains of LSV. a Sequence-alignment variables are mapped onto the surface regions among different LSV strains, with strictly variants highlighted in yellow. b A conformational comparison of βB–βC loops of CPs among LSV2/delta-N48 LSV1 (left), PrV/NωV (middle) and PaV (right). Three distinct βB–βC loops are colored in magenta.

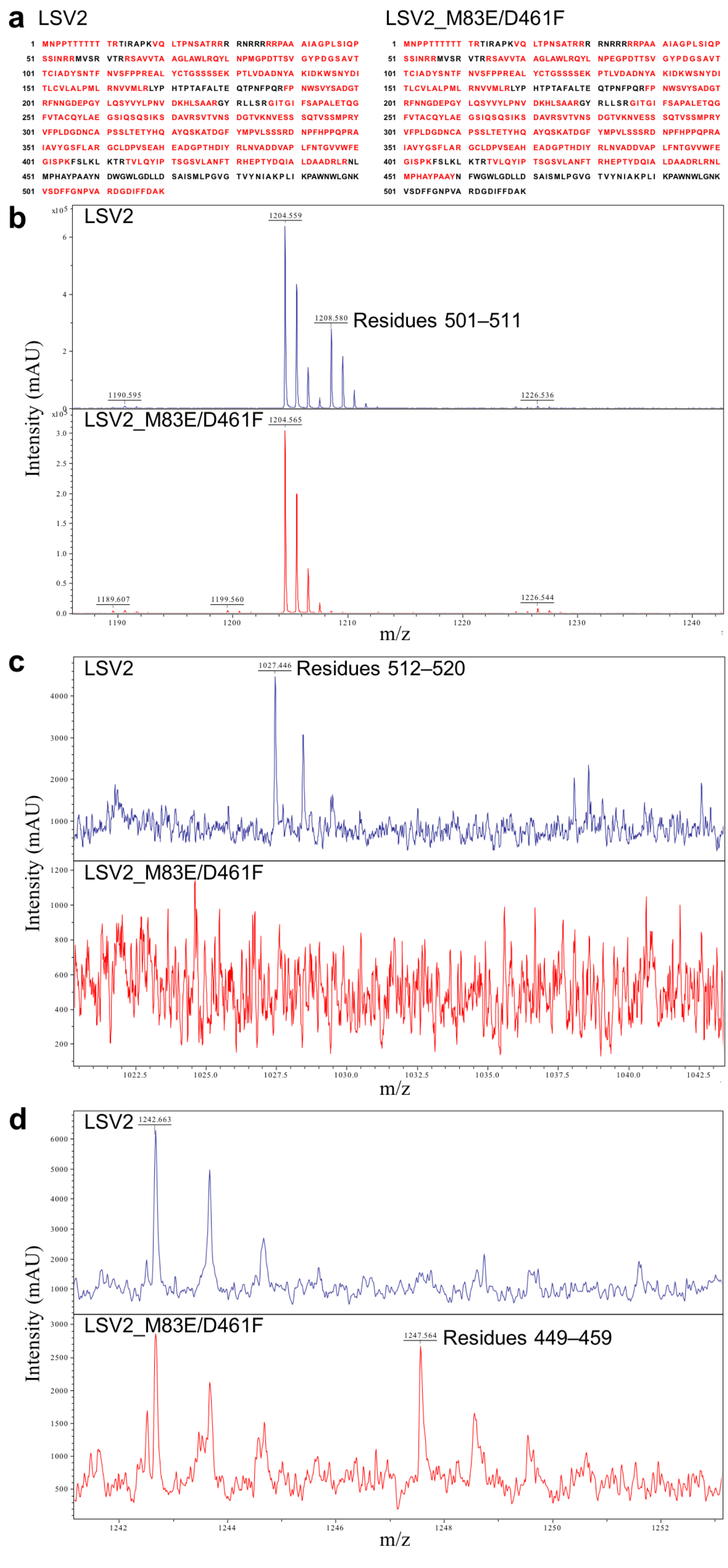

a
LSV2
1 MNPPTTTTTT TRTIRAPKVQ LTPNSATRRR RNRRRRRPAA AIAGPLSIQP
51 SSINRRMVSR VTRRSAVVTA AGLAWLRQYL NPMGPDTTSV GYPDGSAVT
101 TCIADYSNTF NVSFPPREAL YCTGSSSSEK PTLVDADNYA KIDKWSNYDI
151 TLCVLALPML RNVVMLRLYP HTPTAFALTE QTPNFPQRFP NWSVYSADGT
201 RFNNGDEPGY LQSYVYLPNV DKHLSAARGY RLLSRGITGI FSAPALETQG
251 FVTACQYLAE GSIQSQSIKS DAVRSVTVNS DGTVKNVESS SQTVSSMPRY
301 VFPLDGDNCA PSSLTETYHQ AYQSKATDGF YMPVLSSSRD NPFHPPQPRA
351 IAVYGSFLAR GCLDPVSEAH EADGPTHDIY RLNVADDVAP LFNTGVVWFE
401 GISPKFSLKL KTRTVLQYIP TSGSVLANFT RHEPTYDQIA LDAADRLRNL
451 MPHAYPAAYN DWGWLGDLLD SAISMLPGVG TVYNIAKPLI KPAWNWLGNK
501 VSDFFGNPVA RDGDIFFDAK
LSV2_M83E/D461F
1 MNPPTTTTTT TRTIRAPKVQ LTPNSATRRR RNRRRRRPAA AIAGPLSIQP
51 SSINRRMVSR VTRRSAVVTA AGLAWLRQYL NPEGPDTTSV GYPDGSAVT
101 TCIADYSNTF NVSFPPREAL YCTGSSSSEK PTLVDADNYA KIDKWSNYDI
151 TLCVLALPML RNVVMLRLYP HTPTAFALTE QTPNFPQRFP NWSVYSADGT
201 RFNNGDEPGY LQSYVYLPNV DKHLSAARGY RLLSRGITGI FSAPALETQG
251 FVTACQYLAE GSIQSQSIKS DAVRSVTVNS DGTVKNVESS SQTVSSMPRY
301 VFPLDGDNCA PSSLTETYHQ AYQSKATDGF YMPVLSSSRD NPFHPPQPRA
351 IAVYGSFLAR GCLDPVSEAH EADGPTHDIY RLNVADDVAP LFNTGVVWFE
401 GISPKFSLKL KTRTVLQYIP TSGSVLANFT RHEPTYDQIA LDAADRLRNL
451 MPHAYPAAYN FWGWLGDLLD SAISMLPGVG TVYNIAKPLI KPAWNWLGNK
501 VSDFFGNPVA RDGDIFFDAK
b
LSV2
1204.559
1208.580
Residues 501–511
1190.595
1226.536
LSV2_M83E/D461F
1204.565
1189.607
1199.560
1226.544
Intensity (mAU)
m/z
c
LSV2
1027.446
Residues 512–520
LSV2_M83E/D461F
Intensity (mAU)
m/z
d
LSV2
1242.663
LSV2_M83E/D461F
1247.564
Residues 449–459
Intensity (mAU)
m/z

Fig. 16 MS spectra of peptide identified from the LSV2 CP and the double mutant M83E/D461F by using MALTI-TOF MS. a The major fragments were detected for the peptides of LSV2 CP (left) and M83E/D461F (right) treated with trypsin and are shown in red. b MS fragment of residues 501–511 was only obtained in the LSV2 CP (upper) but was not detected in M83E/D461F (lower). c MS fragment of residues 512–520 was only obtained in the LSV2 CP (upper) but was not detected in M83E/D461F (lower). d MS fragment of residues 449–459 was only obtained in M83E/D461F (lower) but was not detected in LSV2 CP (upper).

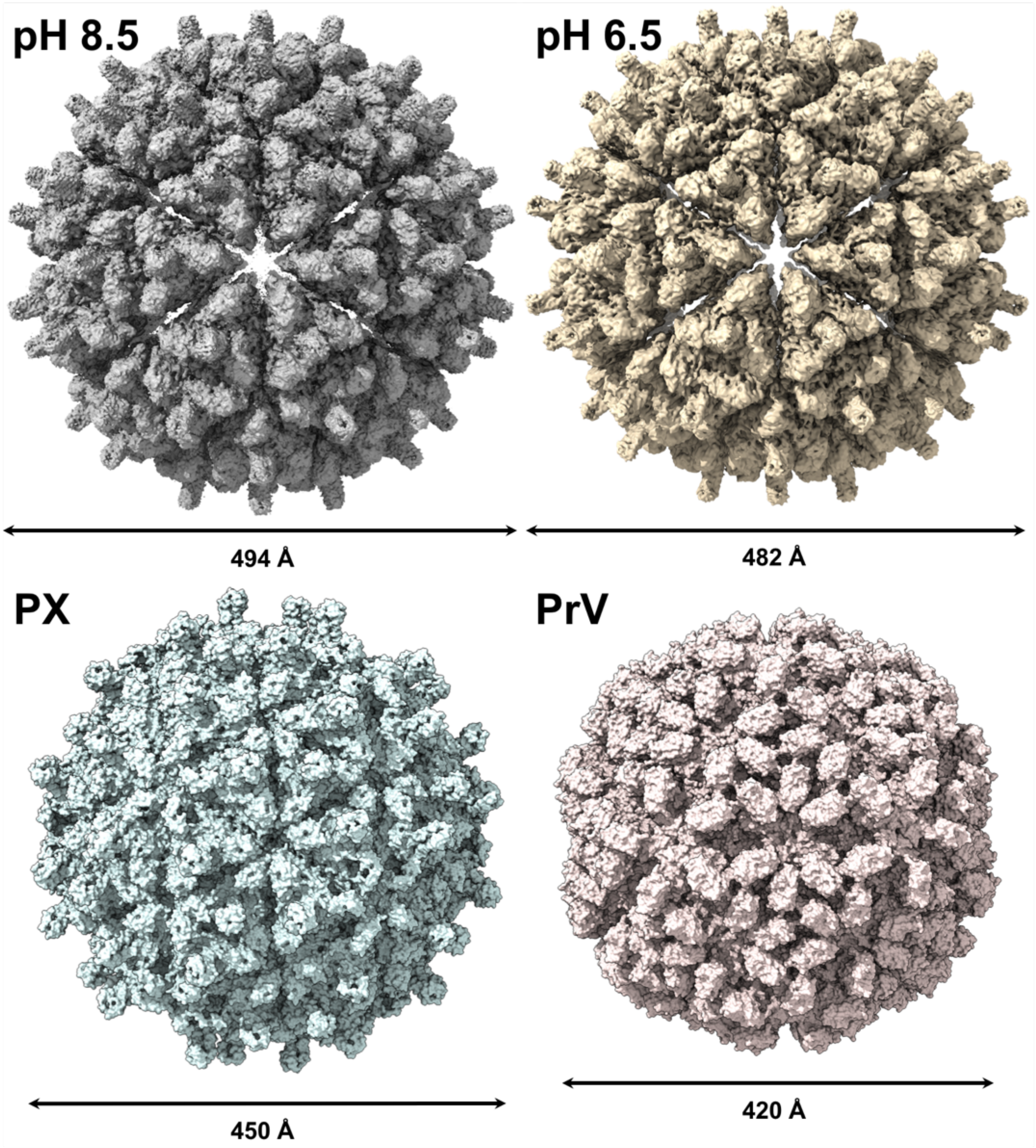

Fig. 17 Particle size comparison of *T*=4 LSV2 VLP and *T*=4 tetravirus PrV. *T*=4 LSV2 VLP (pH 8.5) (upper left), *T*=4 LSV2 VLP (pH 6.5) (upper right), crystal structure of *T*=4 LSV2 VLP (pH 6.5) (lower left) and *T*=4 PrV (pH 7.5) (lower right). The capsid structures of *T*=4 LSV2 VLPs and PrV are shown in gray, gold, cyan and salmon, respectively.

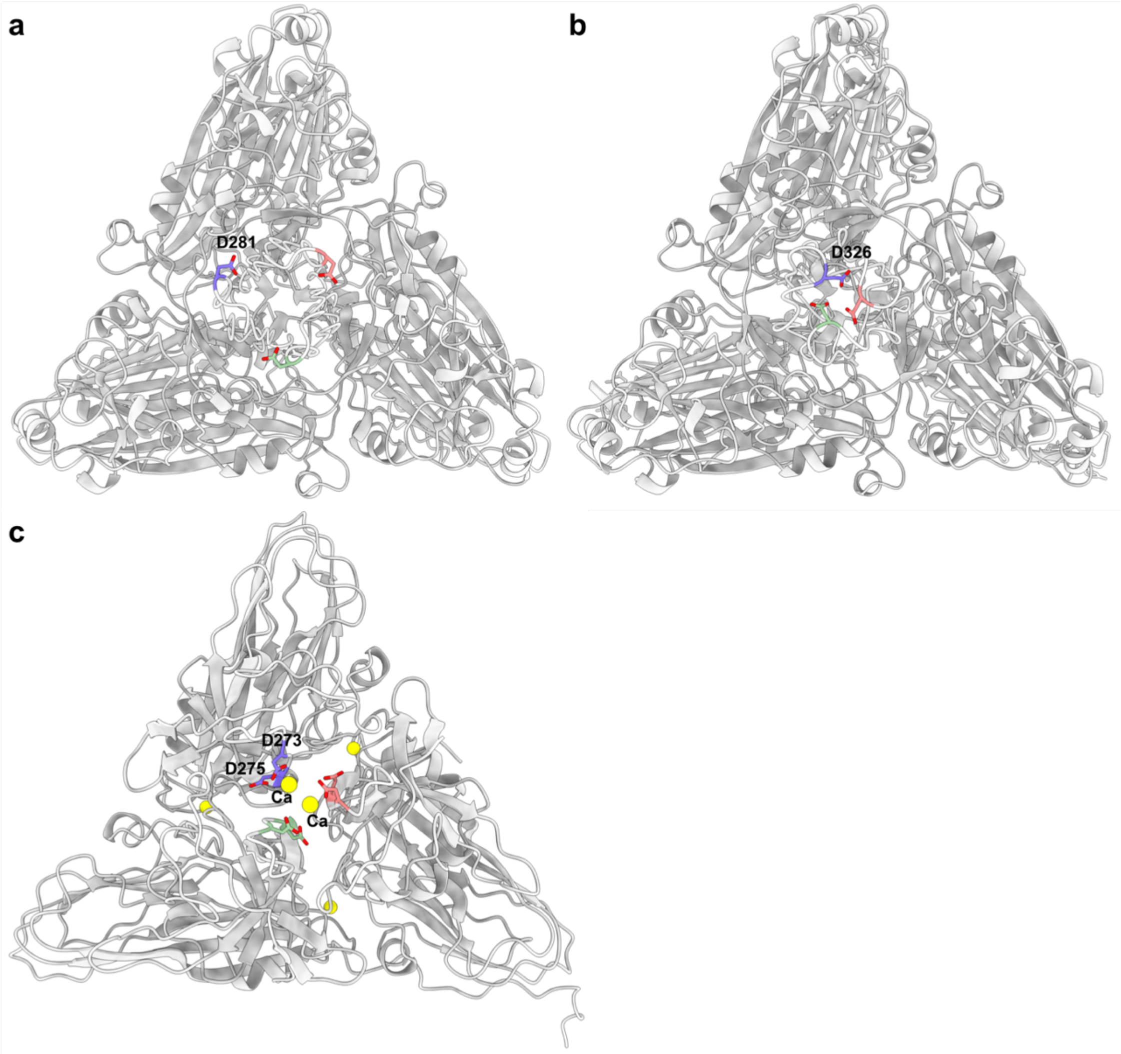

Fig. 18 A comparison of residues Asp on P-domains between LSV and betanodavirus CPs: a LSV2; b delta-N48 LSV1; c GNNV. Residues Asp from subunits A, B and C are shown in purple, red and green sticks, respectively. Ca^{2+} ions are shown as yellow spheres.

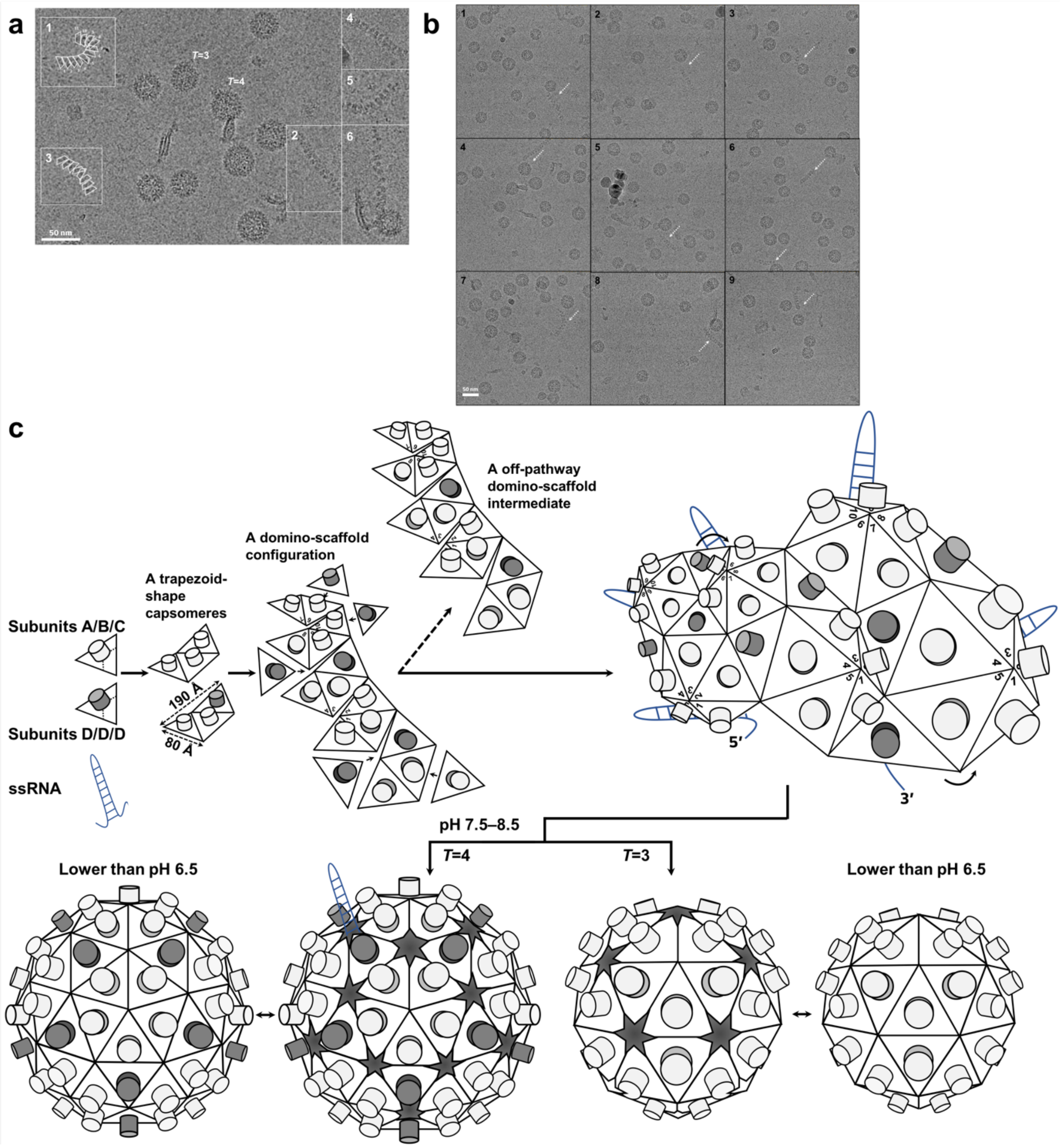

Fig. 19 Self-assembly mechanisms of LSV. a A representative cryo-EM images of some CP subunit organizations in typical domino-scaffold configuration presented together with complete *T*=4 and *T*=3 LSV2 VLP particles, in which the domino-scaffold configurations are clearly shown in six square boxes. Scale bar: 50 nm. 800 independent micrographs gave similar results. Scale bar: 50 nm. b The domino-scaffold configuration appeared often near capsids in cryo-EM images. 800 independent micrographs gave similar results. Scale bar: 50 nm. c Scheme diagrams show the putative self-assembly process of the *T*=4 and *T*=3 LSV complete capsids and the effect of the pH conditions on the dynamic motion of VLP particles. The homo-trimeric capsomeres (A/B/C and D/D/D) form a structurally basic unit for assembly.

One trapezoid-shape capsomere with its shape and size in length of 190 Å and width of 80 Å is composed of three homo-trimeric capsomeres and form one domino-scaffold configuration. The dynamic particle sizes and pore sizes on *T*=4 and *T*=3 LSVs are well correlated with the pH environment. ssRNA can be released from the large shaped holes at I2 axes of *T*=4 LSV.

Table S1 Cryo-EM data collection and icosahedral structure determination statistics

Global refinement cryo-EM structure of *T*=3 LSV2 VLP at pH 6.5
Global refinement cryo-EM structure of *T*=4 LSV2 VLP at pH 6.5
Global refinement cryo-EM structure of *T*=3 LSV2 VLP at pH 7.5
Global refinement cryo-EM structure of *T*=4 LSV2 VLP at pH 7.5
Global refinement cryo-EM structure of *T*=3 LSV2 VLP at pH 8.5
Global refinement cryo-EM structure of *T*=4 LSV2 VLP at pH 8.5
Global refinement cryo-EM structure of *T*=3 delta-N48 LSV1 VLP at pH 6.5
Global refinement cryo-EM structure of *T*=4 delta-N48 LSV1 VLP at pH 6.5 (only map)

	**LSV2 at pH 6.5**		**LSV2 at pH 7.5**		**LSV2 at pH 8.5**		**delta-N48 LSV1 at pH 6.5**	
	***T*=3 VLP (EMD-33370) (PDB 7XPD)**	***T*=4 VLP (EMD-33369) (PDB 7XPB)**	***T*=3 VLP (EMD-33368) (PDB 7XPA)**	***T*=4 VLP (EMD-33190) (PDB 7XGZ)**	***T*=3 VLP (EMD-33372) (PDB 7XPF)**	***T*=4 VLP (EMD-33371) (PDB 7XPE)**	***T*=3 VLP (EMD-33373) (PDB 7XPG)**	***T*=4 VLP (EMD-33384)**
**Data collection**								
EM equipment	Titan Krios		Titan Krios		Titan Krios		Titan Krios	
Voltage (kV)	300		300		300		300	
Cs (mm)	2.7		2.7		2.7		2.7	
Magnification (nominal)	105,000		105,000		105,000		105,000	
Detector	K3		K3		K3		K3	
Pixel size (Å)	0.83		0.83		0.83		0.83	
Electron exposure (e^-/Å^2)	~42		~46		~42		~46	
Exposure time (s)	2.5		2.5		2.5		2.5	
Frames (no.)	40		50		40		50	
Defocus range (µm)	-0.25 ~ -2.72		-0.24 ~ -2.50		-0.27 ~ -2.69		-0.26 ~ -2.71	
**Reconstruction**								
Software	Relion & cryoSPARC		Relion & cryoSPARC		Relion & cryoSPARC		Relion & cryoSPARC	
Symmetry imposed	I		I		I		I	
Micrographs stacks (no.)	10,860		11,245		10,707		13,304	
Final particle images (no.)	41,540	31,723	9,237	30,227	19,860	17,436	40,418	3,425
Map final resolution (Å) †	3.74	3.91	3.33	3.24	3.13	3.32	3.51	4.80
Map sharpening B-factor (Å^2)	145.4	154.8	88.5	112.4	82.7	74.6	130.2	115.5

Table S2 Cryo-EM structure determination and model validation statistics

Focused refinement cryo-EM structure of A/B/C subunits of *T*=4 LSV2 VLP at pH 6.5

Focused refinement cryo-EM structure of D/D/D subunits of *T*=4 LSV2 VLP at pH 6.5

Focused refinement cryo-EM structure of A/B/C subunits of *T*=3 LSV2 VLP at pH 6.5

**LSV2 at pH 6.5**	***T*=4 VLP A/B/C trimer (EMD-33377) (PDB 7XPB)**	***T*=4 VLP D/D/D trimer (EMD-33378) (PDB 7XPB)**	***T*=3 VLP A/B/C trimer (EMD-33379) (PDB 7XPD)**
**Reconstruction**			
Software	Relion & cryoSPARC	Relion & cryoSPARC	Relion & cryoSPARC
Symmetry imposed	C1 (focus refine)	C1 (focus refine)	C1 (focus refine)
Map final resolution (Å) †	2.70	2.73	2.63
Map sharpening B-factor ($Å^2$)	-98.5	-101.5	-94.2
**Atomic modeling**			
Software	Coot & Phenix	Coot & Phenix	Coot & Phenix
Model composition			
Nonhydrogen atoms #	10392	3464	10392
protein residues #	1344	448	1344
ligands #	-	-	-
Map CC (around atoms) *	0.84	0.82	0.83
RMSD bond lengths (Å)	0.008	0.007	0.007
RMSD bond angles (°)	0.734	0.662	0.657
Clash score *	11.27	9.85	13.63
Ramachandran favored (%) *	91.48	91.48	91.18
Ramachandran allowed (%) *	8.52	8.52	8.82
Ramachandran outliers (%) *	0.00	0.00	0.00
Rotamer outliers (%) *	0.00	0.00	0.00
C_β deviations *	0.00	0.00	0.00
MolProbity score *	2.07	2.02	2.16
EMRinger score	3.29	3.85	3.54

†According to FSC=0.143; # Statistics are given for the masked trimer

* According to the criterion of Chen *et al*., 2010[1].

Table S3 Cryo-EM data collection, refinement and validation statistics

Focused refinement cryo-EM structure of A/B/C subunits of *T*=4 LSV2 VLP at pH 7.5

Focused refinement cryo-EM structure of D/D/D subunits of *T*=4 LSV2 VLP at pH 7.5

Focused refinement cryo-EM structure of A/B/C subunits of *T*=3 LSV2 VLP at pH 7.5

**LSV2 at pH 7.5**	***T*=4VLP A/B/C trimer (EMD-33374) (PDB 7XGZ)**	***T*=4 VLP D/D/D trimer (EMD-33375) (PDB 7XGZ)**	***T*=3 VLP A/B/C trimer (EMD-33376) (PDB 7XPA)**
**Reconstruction**			
Software	Relion & cryoSPARC	Relion & cryoSPARC	Relion & cryoSPARC
Symmetry imposed	C1 (focus refine)	C1 (focus refine)	C1 (focus refine)
Map final resolution (Å) †	2.39	2.46	2.60
Map sharpening B-factor ($Å^2$)	-73.2	-78.7	-72.6
**Atomic modeling**			
Software	Coot & Phenix	Coot & Phenix	Coot & Phenix
Model composition			
Nonhydrogen atoms #	10392	3464	10392
protein residues #	1344	448	1344
ligands #	-	-	-
Map CC (around atoms) *	0.86	0.85	0.85
RMSD bond lengths (Å)	0.006	0.004	0.011
RMSD bond angles (°)	0.636	0.624	0.821
Clash score *	9.46	9.12	11.47
Ramachandran favored (%) *	93.42	93.95	92.15
Ramachandran allowed (%) *	6.58	6.05	7.85
Ramachandran outliers (%) *	0.00	0.00	0.00
Rotamer outliers (%) *	4.76	5.03	0.00
C_β deviations *	0.00	0.00	0.00
MolProbity score *	2.44	2.42	2.06
EMRinger score	3.56	3.51	3.54

†According to FSC=0.143; # Statistics are given for the masked trimer

* According to the criterion of Chen *et al*., 2010[1].

Table S4 Cryo-EM data collection, refinement and validation statistics

Focused refinement cryo-EM structure of A/B/C subunits of *T*=4 LSV2 VLP at pH 8.5

Focused refinement cryo-EM structure of D/D/D subunits of *T*=4 LSV2 VLP at pH 8.5

Focused refinement cryo-EM structure of A/B/C subunits of *T*=3 LSV2 VLP at pH 8.5

**LSV2 at pH 8.5**	***T*=4 VLP A/B/C trimer (EMD-33380) (PDB 7XPE)**	***T*=4 VLP D/D/D trimer (EMD-33381) (PDB 7XPE)**	***T*=3 VLP A/B/C trimer (EMD-33382) (PDB 7XPF)**
**Reconstruction**			
Software	Relion & cryoSPARC	Relion & cryoSPARC	Relion & cryoSPARC
Symmetry imposed	C1 (focus refine)	C1 (focus refine)	C1 (focus refine)
Map final resolution (Å) †	2.44	2.50	2.32
Map sharpening B-factor (Å^2)	-62.2	-68.1	-56.3
**Atomic modeling**			
Software	Coot & Phenix	Coot & Phenix	Coot & Phenix
Model composition			
Nonhydrogen atoms #	10392	3464	10392
protein residues #	1344	448	1344
ligands #	-	-	-
Map CC (around atoms) *	0.86	0.85	0.85
RMSD bond lengths (Å)	0.007	0.004	0.004
RMSD bond angles (°)	0.651	0.643	0.598
Clash score *	10.39	7.79	4.48
Ramachandran favored (%) *	91.48	93.27	95.52
Ramachandran allowed (%) *	8.52	6.73	5.16
Ramachandran outliers (%) *	0.00	0.00	0.00
Rotamer outliers (%) *	3.17	4.50	3.35
C_β deviations *	0.00	0.00	0.00
MolProbity score *	2.42	2.36	2.22
EMRinger score	3.34	3.47	3.25

†According to FSC=0.143; # Statistics are given for the masked trimer

* According to the criterion of Chen *et al.*, 2010[1].

Table S5 Cryo-EM data collection, refinement and validation statistics

Focused refinement cryo-EM structure of the A/B/C subunits of *T*=3 delta-N48 LSV1 VLP at pH 6.5

**delta-N48 LSV1 at pH 6.5**	***T*=4 VLP A/B/C trimer**	***T*=4 VLP D/D/D trimer**	***T*=3 VLP A/B/C trimer (EMD-33383) (PDB 7XPG)**
**Reconstruction**			
Software	Relion & cryoSPARC	Relion & cryoSPARC	Relion & cryoSPARC
Symmetry imposed	C1 (focus refine)	C1 (focus refine)	C1 (focus refine)
Map final resolution (Å) †	4.23	4.21	2.58
Map sharpening B-factor ($Å^2$)	-188.8	-174.6	-83.0
**Atomic modeling**			
Software	ND	ND	Coot & Phenix
Model composition			
Nonhydrogen atoms #	ND	ND	10935
protein residues #	ND	ND	1389
Nucleotide#			6
ligands #	ND	ND	-
Map CC (around atoms) *	ND	ND	0.79
RMSD bond lengths (Å)	ND	ND	0.004
RMSD bond angles (°)	ND	ND	0.803
Clash score *	ND	ND	13.66
Ramachandran favored (%) *	ND	ND	94.65
Ramachandran allowed (%) *	ND	ND	5.35
Ramachandran outliers (%) *	ND	ND	0.00
Rotamer outliers (%) *	ND	ND	4.86
C_β deviations *	ND	ND	0.00
MolProbity score *	ND	ND	2.53
EMRinger score	ND	ND	2.75

†According to FSC=0.143; # Statistics are given for the masked trimer

*According to the criterion of Chen *et al.*, 2010[1].

Abbreviation: ND, not determined.

Table S6 Data collection of X-ray crystallography

	T=4 LSV2 VLP
**Data collection**	
Beamline	BL44XU
Wavelength (Å)	0.900
Temperature (K)	110
Space group	$P4_2$
Cell dimensions (Å)	
a	659.96
b	659.96
c	495.74
Resolution (Å)*	30–8.00 (8.28–8.00)
Completeness (%)*	98.6(98.8)
Redundancy*	4.0(3.8)
$\langle I/\sigma_I \rangle$*	2.7(0.9)
R_{sym}(%)†*	30.0(100)

*Values in parentheses are for highest-resolution shell.

†$R_{sym} = \Sigma_h \Sigma_i [| I_i (h) - \langle I(h) \rangle | / \Sigma_h \Sigma_i I_i (h)]$, where I_i is the *i*-th measurement and $\langle I(h) \rangle$ is the weighted mean of all measurements of I(h).

Table S7 Diameter determination of small angle X-ray scattering

Diameter (Å)	SAXS	SAXS with 2 P-domains (~60 Å)
pH 8.5_T=3 LSV2 VLP	434	494
pH 8.5_T=4 LSV2 VLP	389	449
pH 6.5_T=3 LSV2 VLP	422	482
pH 6.5_T=4 LSV2 VLP	378	438

Table S8. A_{260}/A_{280} ratios of LSV2 and delta-N48 LSV1 VLPs

A_{260}/A_{280} ratios of LSV2 and delta-N48 LSV1 VLPs	
LSV2 VLPs (pH 7.5)	1.68 ± 0.2
delta-N48 LSV1 VLPs (pH 6.5)	1.88 ± 0.1

A_{260}/A_{280} ratio reported in the table as the averaged values ± the absolute deviation among the three calculated values.

Source data are provided as a Source Data file.

Reference:

1 Chen, V. B. *et al.* MolProbity: all-atom structure validation for macromolecular crystallography. *Acta Crystallogr. D Biol. Crystallogr.* **66**, 12-21 (2010).
