# Supplementary material for: Structures of honeybee-infecting Lake Sinai virus reveal domain functions and capsid assembly with dynamic motions
Source: Nat Commun. 2023 Feb 1;14:545. doi: 10.1038/s41467-023-36235-3 (PMC9892032; doi:10.1038/s41467-023-36235-3)
Supplement: Supplementary file 3 — Reporting Summary [file 41467_2023_36235_MOESM3_ESM.pdf]

nature portfolio

Corresponding author(s): Chun-Jung Chen; Meng-Chiao Ho

Last updated by author(s): Jan 6, 2023

Reporting Summary

Nature Portfolio wishes to improve the reproducibility of the work that we publish. This form provides structure for consistency and transparency in reporting. For further information on Nature Portfolio policies, see our Editorial Policies and the Editorial Policy Checklist.

Statistics

For all statistical analyses, confirm that the following items are present in the figure legend, table legend, main text, or Methods section.

n/a	Confirmed	
☐	☒	The exact sample size (*n*) for each experimental group/condition, given as a discrete number and unit of measurement
☐	☒	A statement on whether measurements were taken from distinct samples or whether the same sample was measured repeatedly
☒	☐	The statistical test(s) used AND whether they are one- or two-sided<br>*Only common tests should be described solely by name; describe more complex techniques in the Methods section.*
☒	☐	A description of all covariates tested
☒	☐	A description of any assumptions or corrections, such as tests of normality and adjustment for multiple comparisons
☐	☒	A full description of the statistical parameters including central tendency (e.g. means) or other basic estimates (e.g. regression coefficient) AND variation (e.g. standard deviation) or associated estimates of uncertainty (e.g. confidence intervals)
☒	☐	For null hypothesis testing, the test statistic (e.g. *F*, *t*, *r*) with confidence intervals, effect sizes, degrees of freedom and *P* value noted<br>*Give P values as exact values whenever suitable.*
☒	☐	For Bayesian analysis, information on the choice of priors and Markov chain Monte Carlo settings
☒	☐	For hierarchical and complex designs, identification of the appropriate level for tests and full reporting of outcomes
☒	☐	Estimates of effect sizes (e.g. Cohen's *d*, Pearson's *r*), indicating how they were calculated

Our web collection on statistics for biologists contains articles on many of the points above.

Software and code

Policy information about availability of computer code

Data collection	EPU-2.7.0; SerialEM v3.7; HKL2000; NSRRC TPS13A1 SAXS DR-GUI
Data analysis	CTFFIND v4; CTFFIND v4.1; cisTEM v1; MotionCor2 v1.3.0; Relion 3.0; cryoSPARC v3.2; PHENIX software suite; UCSF Chimera v1.15; UCSF ChimeraX v1.2; PyMOL v1.2r3pre; Coot v0.9; ATSAS package; SASview 5.0.4; Phaser v2.7; FlexControl v3.4; FlexAnalysis v3.4; Biotools v3.2; Mascot server online

For manuscripts utilizing custom algorithms or software that are central to the research but not yet described in published literature, software must be made available to editors and reviewers. We strongly encourage code deposition in a community repository (e.g. GitHub). See the Nature Portfolio guidelines for submitting code & software for further information.

Data

Policy information about availability of data

All manuscripts must include a data availability statement. This statement should provide the following information, where applicable:

- Accession codes, unique identifiers, or web links for publicly available datasets
- A description of any restrictions on data availability
- For clinical datasets or third party data, please ensure that the statement adheres to our policy

The consensus DNA sequence coding for the LSV1 CP (GenBank accession No. ASS83276.1) and LSV2 CP (GenBank accession No. AEH26188.1). Cryo-EM maps in this study have been deposited to the Electron Microscopy Data Bank (EMDB) under accession codes EMD-33190 (T=4 LSV2 VLP at pH 7.5, global refinement), EMD-33368 (T=3 LSV2 VLP at pH 7.5, global refinement), EMD-33369 (T=4 LSV2 VLP at pH 6.5, global refinement), EMD-33370 (T=3 LSV2 VLP at pH 6.5, global

refinement), EMD-33371 (T=4 LSV2 VLP at pH 8.5, global refinement), EMD-33372 (T=3 LSV2 VLP at pH 8.5, global refinement), EMD-33384 (T=4 delta-N48 LSV1 VLP at pH 6.5, global refinement), EMD-33373 (T=3 delta-N48 LSV1 VLP at pH 6.5, global refinement), EMD-33374 (A/B/C trimer of T=4 LSV2 VLP at pH 7.5, focused refinement), EMD-33375 (D/D/D trimer of T=4 LSV2 VLP at pH 7.5, focused refinement), EMD-33376 (A/B/C trimer of T=3 LSV2 VLP at pH 7.5, focused refinement), EMD-33377 (A/B/C trimer of T=4 LSV2 VLP at pH 6.5, focused refinement), EMD-33378 (D/D/D trimer of T=4 LSV2 VLP at pH 6.5, focused refinement), EMD-33379 (A/B/C trimer of T=3 LSV2 VLP at pH 6.5, focused refinement), EMD-33380 (A/B/C trimer of T=4 LSV2 VLP at pH 8.5, focused refinement), EMD-33381(D/D/D trimer of T=4 LSV2 VLP at pH 8.5, focused refinement), EMD-33382 (A/B/C trimer of T=3 LSV2 VLP at pH 8.5, focused refinement), EMD-33383 (A/B/C trimer of T=3 LSV2 VLP at pH 6.5, focused refinement). Their corresponding atomic models have been deposited to the RCSB Protein Data Bank (PDB) under accession numbers 7XGZ, 7XPA, 7XPB, 7XPD, 7XPE, 7XPF and 7XPG, respectively.

Human research participants

Policy information about studies involving human research participants and Sex and Gender in Research.

Reporting on sex and gender	N/A
Population characteristics	N/A
Recruitment	N/A
Ethics oversight	N/A

Note that full information on the approval of the study protocol must also be provided in the manuscript.

Field-specific reporting

Please select the one below that is the best fit for your research. If you are not sure, read the appropriate sections before making your selection.

☒ Life sciences ☐ Behavioural & social sciences ☐ Ecological, evolutionary & environmental sciences

For a reference copy of the document with all sections, see nature.com/documents/nr-reporting-summary-flat.pdf

Life sciences study design

All studies must disclose on these points even when the disclosure is negative.

Sample size	For cryo-EM data, sufficient sample sizes, including number of images and extracted particles, were determined by collecting enough data until we could obtain high-resolution reconstructed maps (estimated by Fourier shell correlation). X-ray crystallographic data was collected on the highest-resolution crystal after screening more than 20 crystals. Small-angle X-ray scattering data was collected on independent samples twice. Absorption assay was performed in three replicates.
Data exclusions	No data were excluded from the analyses
Replication	All cryo-EM data in different conditions were calculated using standard procedures at highest resolution without replications. X-ray crystallographic data on more than 20 crystals (20 replicates), including the highest-resolution crystal, were examined and gave the same space group and unit cell. Small-angle X-ray scattering data was collected on independent samples twice and gave a similar result. Absorption assay was performed in three replicates, giving the similar result.
Randomization	The datasets were randomly split into two halves which were refined independently in cryoSPARC. The overall resolution was estimated with a criterion Fourier Shell Correlation (FSC) = 0.143; the local resolution were also calculated in cryoSPARC
Blinding	Blinding is not applicable to this study. Particle assignment to every half-sets and the corresponding resolution estimation were performed automatically by processing software packages.

Reporting for specific materials, systems and methods

We require information from authors about some types of materials, experimental systems and methods used in many studies. Here, indicate whether each material, system or method listed is relevant to your study. If you are not sure if a list item applies to your research, read the appropriate section before selecting a response.

Materials & experimental systems

n/a	Involved in the study
☒	☐ Antibodies
☒	☐ Eukaryotic cell lines
☒	☐ Palaeontology and archaeology
☒	☐ Animals and other organisms
☒	☐ Clinical data
☒	☐ Dual use research of concern

Methods

n/a	Involved in the study
☒	☐ ChIP-seq
☒	☐ Flow cytometry
☒	☐ MRI-based neuroimaging
